# Generalized Structured Component Analysis Accommodating Convex Components: A Knowledge-Based Multivariate Method with Interpretable Composite Indexes

**DOI:** 10.1007/s11336-023-09944-3

**Published:** 2024-02-16

**Authors:** Gyeongcheol Cho, Heungsun Hwang

**Affiliations:** 1 Department of Psychology, The Ohio State University, 1827 Neil Avenue, Columbus, OH 43210 USA; 2 Department of Psychology, McGill University, Montreal, Canada

**Keywords:** generalized structured component analysis, convex component, multivariate analysis, composite index, interpretability

## Abstract

Generalized structured component analysis (GSCA) is a multivariate method for examining theory-driven relationships between variables including components. GSCA can provide the deterministic component score for each individual once model parameters are estimated. As the traditional GSCA always standardizes all indicators and components, however, it could not utilize information on the indicators’ scale in parameter estimation. Consequently, its component scores could just show the relative standing of each individual for a component, rather than the individual’s absolute standing in terms of the original indicators’ measurement scales. In the paper, we propose a new version of GSCA, named *convex GSCA*, which can produce a new type of unstandardized components, termed convex components, which can be intuitively interpreted in terms of the original indicators’ scales. We investigate the empirical performance of the proposed method through the analyses of simulated and real data.

Generalized structured component analysis (GSCA; Hwang & Takane, 2004; 2014) is a multivariate method that allows for specifying and testing path-analytic relationships between observed variables and components (i.e., weighted sums of observed variables). Observed variables forming components are called composite indicators (Bollen and Bauldry, 2011). Given a theory-driven model, GSCA constructs components from composite indicators such that the components can explain the total variances of all dependent variables in the model as much as possible.

As in many component analysis techniques, GSCA has typically assumed that all components and indicators were standardized to have zero means and unit variances. This traditional, standardized version of GSCA shall be called GSCA$_{std}$ hereafter. GSCA$_{std}$ begins by standardizing indicators prior to estimating parameters and updates component weights in such a way that they produce standardized components during the estimation process. Such standardization can be useful for the interpretation and comparison of GSCA$_{std}$’s estimates because the GSCA$_{std}$ model is equivalent to a system of multiple regression equations for standardized components and indicators, indicating that its loadings and path coefficients can be interpreted as standardized regression coefficients.

Nonetheless, the conventional standardization of components makes it difficult to interpret *component scores* in terms of the original indicators’ measurement scales. The standardized component score for an individual merely shows the individual’s relative location to the other individuals in the sample and the absolute score itself is not interpretable. This is less attractive to researchers who are interested in the absolute level of a component for each individual. For example, if a standardized component is used to measure the level of life satisfaction, an individual’s component score can inform whether s/he has a relatively lower or higher level of life satisfaction than the others. However, it cannot tell exactly what the level of her/his life satisfaction is, reflecting whether s/he is satisfied or dissatisfied with her/his life.

Moreover, if indicators for each component are measured on the same scale, which is often observed in practice, standardizing the indicators may not be recommended because it can eliminate “the natural and relevant variability present” (Naik and Khattree, 1996) in each of the indicators, forcing them to have the same variance, although their variances may not be the same in reality. For illustration, suppose that we made two versions of test batteries to assess children’s intelligence, both of which were measured on a 0 to 100 scale. Three children took these tests and obtained {49, 50, 51} for Test 1 and {0, 50, 100} for Test 2. The results show that Test 1 almost fails to differentiate the children’s intelligence levels, whereas Test 2 differentiates their intelligence level very well, indicating that the difference in score variability between the two tests is interpretable and contains meaningful information. However, when we standardize these scores, such information disappears since both score sets become identical (i.e., {–1, 0, 1} ). If GSCA$_{std}$ is applied to the tests, the same standardized weight values (i.e., .5) will be assigned to the two tests.

To obtain unstandardized component scores from original indicators, GSCA$_{std}$ applies an additional rescaling of weight estimates after convergence (Hwang & Takane, 2014, Chapter 2). As will be discussed in more detail in Sect. 1, each indicator’s weight estimate is rescaled by dividing it by the indicator’s standard deviation. Subsequently, unstandardized component scores are obtained by pre-multiplying the rescaled weights by their indicators’ original scores.

However, this rescaling procedure has two issues. Firstly, the procedure is carried out while keeping the variances of components fixed to one. Thus, the variances of the resultant unstandardized components are likely to be different from those of the original indicators, so that it is not guaranteed that the unstandardized component scores would vary within the same range of the original indicators. Secondly, the rescaling procedure tends to have indicators with relatively small variances influence the construction of their unstandardized component more heavily. In the above example, as the sample standard deviations of the two test batteries were 1 and 50, the unstandardized weights obtained from this ad-hoc rescaling procedure would be .5 and .01 for Tests 1 and 2, respectively. This indicates that Test 1 is 50 times more influential for forming children’s unstandardized component scores than Test 2, even though Test 2 differentiates children’s intelligence levels much better than Test 1. In Sect. 1, we will explain why this issue occurs in the rescaling procedure.

To address these issues, we propose a different version of GSCA, named *convex GSCA* or GSCA$_{cvx}$ for short, which can estimate unstandardized components of original indicators. Specifically, GSCA$_{cvx}$ obtains an unstandardized component as a convex combination of original indicators, termed a *convex component*, if the indicators for the component have the same measurement scale. A convex combination of a set of vectors refers to a special linear combination whose weights are non-negative and summed up to one (Lay et al., 2015, Chapter 8). As will be shown in Sect. 2, a convex component’s scores are within the same range of its indicators’ scores. This property of the convex component facilitates the interpretation of its component scores with reference to the indicators’ scales. Moreover, GSCA$_{cvx}$ avoids the unnecessary standardization of indicators when they are on the same measurement scales, allowing for utilizing information on their variances in parameter estimation.

The remaining sections of the paper are organized as follows. In Sect. 1, we briefly describe GSCA$_{std}$ and explain its ad-hoc procedure of computing unstandardized components and the procedure’s limitation. In Sect. 2, we introduce a convex component and explain its six properties. In Sect. 3, we present the GSCA$_{cvx}$ model that accommodates convex components and propose an iterative algorithm for estimating model parameters. We also provide a set of overall goodness-of-fit and cross-validation indexes for model evaluation and comparison. In Sect. 4, we conduct a Monte-Carlo simulation study to examine GSCA$_{cvx}$’s parameter recovery. In Sect. 5, we apply GSCA$_{cvx}$ to real data to demonstrate its practical usefulness. In Sect. 6, we summarize the previous sections and discuss the method’s implications and prospective extensions.

## Traditional GSCA with Standardized Variables

### Model and Parameter Estimation

GSCA$$_{std}$$ involves three sub-models—weighted relation, component measurement, and structural models (Hwang and Takane, 2004, 2014). Let **z**$$_{std} =$$ [z$$_{std,1}$$, z$$_{std,2}$$, $$\cdots $$, z$$_{std,J}$$]$$^\prime $$ denote a *J* by 1 random vector of standardized indicators, where z$$_{std,j}$$ is the *j*th standardized indicator, i.e., *E*(z$$_{std,j}) =$$ 0 and *var*(z$$_{std,j}) =$$ 1 ($$j =$$ 1, 2, $$\cdots $$, *J*). The mean of **z**$$_{std}$$ is a zero vector and the correlation matrix of **z**$$_{std}$$ is denoted by $$\varvec{\Sigma } _{std}$$. Let $$\varvec{\upgamma }_{std} =$$ [$$\upgamma _{std,1}$$, $$\upgamma _{std,2}$$, $$\cdots $$, $$\upgamma _{std,P}]^{\prime }$$ denote a *P* by 1 random vector of standardized components, where $$\upgamma _{std,p}$$ is the *p*th standardized component, i.e., $$E(\upgamma _{std,p}) =$$ 0, *var*($$\upgamma _{std,p}) =$$ 1 ($$p =$$ 1, 2, $$\cdots $$, *P*). Let **W**$$_{std}$$ denote a *J* by *P* matrix consisting of component weights assigned to indicators. Let **C**$$_{std}$$ denote a *P* by *J* matrix of loadings relating components to indicators. Let **B**$$_{std}$$ denote a *P* by *P* matrix of path coefficients relating components to each other. Let $${\varvec{\upxi }} =$$ [$$\upxi _{{1}}$$, $$\upxi _{{2}}$$, $$\cdots $$, $$\upxi _{J}]^{\prime }$$ denote a *J* by 1 random vector of errors in the component measurement model, where $$\upxi _{j}$$ is an error for the *j*th indicator. Let $${\varvec{\upzeta }}$$ $$=$$ [$$\upzeta _{{1}}$$, $$\upzeta _{{2}}$$, $$\cdots $$, $$\upzeta _{P}]^{\prime }$$ denote a *P* by 1 random vector of errors in the structural model, where $$\upzeta _{p}$$ is an error for the *p*th component. The three sub-models of GSCA$$_{std}$$ are expressed as follows.

1 $$\begin{aligned} {\varvec{\upgamma }}_{std}\equiv &  {{{\textbf {W}}}}_{std}'{{{\textbf {z}}}}_{std} \,(\text {weighted relation model}) \end{aligned}$$

2 $$\begin{aligned} {{{\textbf {z}}}}_{std}= &  {{{\textbf {C}}}}_{std}'{\varvec{\upgamma }}_{std} + {\varvec{\upxi }} \,(\text {component measurement model}) \end{aligned}$$

3 $$\begin{aligned} {\varvec{\upgamma }}_{std}= &  {{{\textbf {B}}}}_{std}'{\varvec{\upgamma }}_{std} + {\varvec{\upzeta }} \,(\text {structural model}). \end{aligned}$$

The weighted relation model (1) shows that (standardized) components are defined as a linear combination of standardized indicators. The component measurement and structural models (2) and (3) express the directional relationships between the indicators and components and those among the components, respectively. As (2) and (3) can be seen as systems of linear regression equations, their model parameters, including loadings and path coefficients, can be interpreted in the same manner as standardized regression coefficients. The three sub-models are combined into the following equation,

4 $$\begin{aligned} &  \text {[}{{{\textbf {z}}}}_{std};{\varvec{\upgamma }}_{std}]=[{{{\textbf {C}}}}_{std},{{{\textbf {B}}}}_{std}\text {]}^\prime {\varvec{\upgamma }}_{std}+\left[ {\varvec{\upxi };\varvec{\upzeta }} \right] \nonumber \\ &  \quad {\leftrightarrow }\text {[}{{{\textbf {I}}}}_{J},{{{\textbf {W}}}}_{std}\text {]}^\prime {{{\textbf {z}}}}_{std}=[ {{{\textbf {C}}}}_{std},{{{\textbf {B}}}}_{std}]'{{{{\textbf {W}}}}_{std}'\textbf{z}}_{std}+\left[ {\varvec{\upxi };\varvec{\upzeta }} \right] \nonumber \\ &  \quad {\leftrightarrow }{{{{\textbf {V}}}}_{std}'\textbf{z}}_{std}={{{\textbf {A}}}}_{std}'{{{\textbf {W}}}}_{std}'{{{\textbf {z}}}}_{std}+\textbf{e}, \end{aligned}$$

where **I**$_{J}$ is the identity matrix of order *J*, ${{{\textbf {V}}}}_{std} \equiv$ [**I**$_{J}$, ${{{\textbf {W}}}}_{std}$], ${{{\textbf {A}}}}_{std} \equiv$ $\text {[}{{{\textbf {C}}}}_{std},{{{\textbf {B}}}}_{std}\text {]}$, **e** $\equiv$ [${\varvec{\upxi }}$; ${\varvec{\upzeta }}$] and a semicolon within brackets is an operator to vertically concatenate two vectors in the array. The equation (4) is called the GSCA$_{std}$ model.

Let **1**$_{Q}$ denote a column vector of *Q* ones. Let *SS*(**X**) $\equiv$ *tr*(**X**$^{\prime }$ **X**) for any matrix **X**. Let *vecdiag*() denote an operator that returns a column vector stacking the diagonal elements of a square matrix one below another. GSCA$_{std}$ estimates model parameters (${{{\textbf {W}}}}_{std}$ and ${{{\textbf {A}}}}_{std})$ by minimizing the following objective function

5 $$\begin{aligned} &  f_{std}({{{\textbf {W}}}}_{std},{{{\textbf {A}}}}_{std})\nonumber \\ &  \quad =tr(E({{{\textbf {e}}}}_{std}{{{\textbf {e}}}}_{std}'))\nonumber \\ &  \quad =E(SS([{{{\textbf {z}}}}_{std};{\varvec{\upgamma }}_{std}]'-{{{\textbf {z}}}}_{std}'{{{\textbf {W}}}}_{std}{{{\textbf {A}}}}_{std}),\nonumber \\ &  \quad =E(SS({{{\textbf {z}}}}_{std}'([{{{\textbf {I}}}}_{J},{{{\textbf {W}}}}_{std}]-{{{\textbf {W}}}}_{std}{{{\textbf {A}}}}_{std}))\nonumber \\ &  \quad =tr (({{{\textbf {V}}}}_{std}-{{{\textbf {W}}}}_{std}{{{\textbf {A}}}}_{std})'{\varvec{\Sigma }}_{std}({{{\textbf {V}}}}_{std}-{{{\textbf {W}}}}_{std}{{{\textbf {A}}}}_{std})) \end{aligned}$$

subject to *vecdiag*(${{{\textbf {W}}}}_{std}'{\varvec{\Sigma }}_{std}{{{\textbf {W}}}}_{std})$ $=\,$ **1**$_{p}$. Thus, GSCA$_{std}$ estimates the model parameters by minimizing the sum of error variances for all variables in the model given ${\varvec{\Sigma }}_{std}$. In general, ${\varvec{\Sigma }}_{std}$ is replaced with the sample correlation matrix of indicators, denoted by **S**$_{std}$. The objective function (5) also shows that GSCA$_{std}$ aims to create components that explain the total variances of variables in the model rather than their covariances, as with PCA or other component-based methods. The error terms in the GSCA$_{std}$ model are not considered independent entities that cause the variation of indicators but simply treated as residuals that are unexplained by independent components. Thus, GSCA$_{std}$ typically makes no assumptions about the correlation structure of the error terms of indicators, leaving them freely correlated. This is distinct from the common factor model, where the error terms are typically assumed to be uncorrelated. Nonetheless, no error covariances between different blocks of indicators may be assumed in some special cases of GSCA (Cho et al., 2020, 2022).

Note that (1) defines a component as a weighted sum of indicators, which is also the case in PCA. However, this equation itself is not identified because there would exist infinitely different ways of deciding the component weights. Thus, we need a certain rule or criterion to determine the component weights. PCA’s criterion is one of the most widely used ones in statistics that the weights are to be determined in such a way that their corresponding components explain the maximum total variance of the indicators. The regression coefficients of indicators on their component are (component) loadings. These relationships between components and their indicators are expressed in the component measurement model (2). Thus, GSCA can have confirmatory PCA (Takane, Kiers, & de Leeuw, 1995) as a special case when it considers (1) and (2) only.

As the minimization problem (5) cannot be solved in closed form, an alternating least squares (ALS) algorithm was developed for iteratively finding the minimum point of (5). In the ALS algorithm, **W**$_{std}$ and **A**$_{std}$ are updated alternately with the other fixed until the difference in (5) between consecutive iterations decreases beyond a pre-specified tolerance level (e.g., 10$^{-\text {5}})$ (see Hwang & Takane, 2014, Chapter 2, for a full description of the ALS algorithm). Let ${\varvec{{\widehat{\Gamma }}}}_{std}$ denote an *N* by *P* matrix of the standardized score estimates of components, ${{{\textbf {D}}}}_{std}$ denote an *N* by *J* matrix of the standardized scores of indicators, and *N* is the number of cases in the sample. Let us suppose that we obtain the estimates of **W**$_{std}$ and **A**$_{std}$ that minimize (5), denoted by ${\widehat{{\textbf {W}}}}_{std}$ and ${\widehat{{{\textbf {A}}}}}_{std}$. Then, a matrix of standardized component scores is obtained by

6 $$\begin{aligned} {\varvec{{\widehat{\Gamma }}}}_{std}\,\,{\equiv }\,\,{{{\textbf {D}}}}_{std}{\widehat{{\textbf {W}}}}_{std}. \end{aligned}$$

### Unstandardized Weight Estimates in GSCA $_{std}$

Let **D**$\,{=}\,{{{\textbf {1}}}}_{N}{\varvec{{\hat{\upmu }}}}'\, +\, {{{\textbf {D}}}}_{std}{\varvec{{\widehat{\Delta }}}}_{\text {z}}$ denote an *N* by *J* matrix of the unstandardized scores of indicators, where ${\varvec{{\hat{\upmu }}}}$ is a *J* by 1 sample mean vector and ${\varvec{{\widehat{\Delta }}}}_{\text {z}}$ is a diagonal matrix whose entries are sample standard deviations of unstandardized indicators. Conventionally, unstandardized component means are subsequently computed by transforming ${\widehat{{\textbf {W}}}}_{std}$ as follows. As it follows from (6) that

7 $$\begin{aligned} &  {\varvec{{\widehat{\Gamma }}}}_{std}=({{{\textbf {D}}}}-\textbf{1}_{N}{\varvec{{\hat{\upmu }}}}'){\varvec{\widehat{\Delta }}}_{\text {z}}^{{-1}}{{{\widehat{{\textbf {W}}}}}}_{std} \nonumber \\ &  {{{\leftrightarrow 1}}}_{N}{\varvec{\hat{\upmu }}}'{\varvec{\widehat{\Delta }}}_{\text {z}}^{{-1}}{{\widehat{{\textbf {W}}}}}_{std}+{\varvec{{\widehat{\Gamma }}}}_{std}{=\textbf{D}}{\varvec{\widehat{\Delta }}}_{\text {z}}^{{-1}}{\widehat{{\textbf {W}}}}_{std} \nonumber \\ &  {{{\leftrightarrow 1}}}_{N}{\varvec{\hat{\upmu }}}'{\widehat{{\textbf {W}}}}_{uni}+{\varvec{\widehat{\Gamma }}}_{std}{=\textbf{D}}{\widehat{{\textbf {W}}}}_{uni}, \end{aligned}$$

where ${\widehat{{\textbf {W}}}}_{uni} \equiv {\varvec{\widehat{\Delta }}}_{\text {z}}^{{-1}}{\widehat{{\textbf {W}}}}_{std}$, GSCA$_{std}$ computes unstandardized component scores, denoted here by ${\varvec{{\widehat{\Gamma }}}}_{uni}$, as ${\varvec{{\widehat{\Gamma }}}}_{uni}\,\equiv \,\,$ **D**${\widehat{{\textbf {W}}}}_{uni}$ (Hwang & Takane, 2014, p. 26).

As shown in the last line of (7), however, ${\varvec{{\widehat{\Gamma }}}}_{uni}$ can be simply seen as a variant of standardized component scores whose means are only relocated *a posteriori* by ${{{\textbf {1}}}}_{N}{\varvec{\hat{\upmu }}}{'{\widehat{{\textbf {W}}}}}_{uni}$ in that ${\varvec{\widehat{\Gamma }}}_{std}$ remains standardized irrespective of the sample variances of the original indicators. Consequently, it is not guaranteed that the scores of ${\varvec{{\widehat{\Gamma }}}}_{uni}$ are within the same range of the unstandardized scores of their indicators, which will be empirically shown in Sect. 5. Also, as illustrated in the introduction, GSCA$_{std}$ tends to assign smaller unstandardized weights to original indicators with relatively large variances in forming ${\varvec{{\widehat{\Gamma }}}}_{uni}$. That is because minimizing (5) involves imposing a relatively large penalty on an original indicator with a relatively large variance, which is shown in Appendix 1. This disproportionate penalization for original indicators can inadvertently amplify the influence of an original indicator with a small variance on GSCA$_{std}$’s parameter estimation. Such an approach could be deemed unsuitable when one aims to obtain an unstandardized component of original indicators on a single scale.

## Convex Component and Its Six Properties

Let $\upgamma _{p}$ denote the *p*th component ($p =$ 1, 2, $\cdots$, *P*) that is assumed to have the mean $\uptau _{p}$ and variance $\upphi _{p}$. Let **z**$_{p}$ denote a $J_{p}$ by 1 vector of indicators for $\upgamma _{p}$, where $J_{p}$ is the number of indicators for $\upgamma _{p}$. We call the vector **z**$_{p}$ *a block of* *indicators* for $\upgamma _{p}$, which is assumed to have the mean vector ${\varvec{\upmu }}_{p}$ and covariance matrix $\varvec{\Sigma }_{p}$. Let **w**$_{p}$ denote a $J_{p}$ by 1 vector of weights for **z**$_{p}$. Let **0**$_{k\times l}$ denote a *k* by *l* matrix of zeros, where *k* and *l* are any scalars. If $\upgamma _{p}$ is defined as a convex component, it can be expressed as

8 $$\begin{aligned} \upgamma _{p} \equiv {{\textbf {w}}}_{p}'{{\textbf {z}}}_{p} \text { subject to } {{\textbf {w}}}_{p}'{{\textbf {1}}}_{Jp} = 1 \text { and }{{\textbf {w}}}_{p} \ge {{\textbf {0}}}_{J \times 1}. \end{aligned}$$

A convex component has six useful properties as follows.

### Proposition 1

*A* convex component has scores within the range of its indicators’ scores.

### Proposition 2

Each score of a convex component corresponds to a component score of an individual whose scores for indicators are all the same as the component score.

### Proposition 3

The mean of a convex component is not fixed to zero but is determined by weights within the range of its indicators’ means.

### Proposition 4

The standard deviation of a convex component is not fixed to one but is determined by weights within the range from 0 to the maximum standard deviation of its indicators.

### Proposition 5

Given a linearly independent set of indicators’ scores, a set of convex component scores has a unique set of weights that are nonnegative and summed up to one.

### Proposition 6

The path coefficient of a convex component on an outcome variable indicates the expected amount of change in the outcome variable for a unit change in each indicator of the convex component while holding other variables fixed.

We provide proofs for the six propositions in Appendix 2. The first four properties make a convex component’ scores, mean, and standard deviation interpretable with reference to its indicators’ scale when its indicators are on the same scale. The fifth property allows interpreting weight parameters as the contribution rates of indicators to forming their component. The last property allows for interpreting the path coefficient of a convex component with respect to its indicators’ scale. We here illustrate these properties with an example of (major) depression.

Let us assume that depression can be represented by a convex component ($$\upgamma )$$ with three symptom-related indicators (z$$_{\text {1}}$$ $$= $$ depressed affect, z$$_{\text {2}}$$ $$= $$ somatic discomfort, and z$$_{\text {3}}$$ $$= $$ interpersonal problem), which are commonly rated on a seven-point Likert scale (0 $$=$$ “none”, 1 $$=$$ “minimal”, 2 $$=$$ “mild”, 3 $$=$$ “moderate”, 4 $$=$$ “moderately severe”, 5 $$=$$ “severe”, and 6 $$=$$ “extremely severe”). It is generally considered safe to treat ordinal variables with five or more categories as continuous (Johnson and Creech, 1983; Norman, 2010; Sullivan and Artino, 2013; Zumbo and Zimmerman, 1993). Then, this depression component serves as a summary index whose score indicates the overall severity level of the three depressive symptoms for each individual. Specifically, once weight parameters are estimated, a score set of depression component is obtained given a dataset of its indicators. Proposition 1 indicates that all individuals’ scores of depression component will be within the range of the measurement scale of its indicators (e.g., [0, 6]). Proposition 2 implies that each individual’s score of depression component within the range can be interpreted as the depression level of an individual whose indicators’ scores are all the same as the depression component score. For example, if a patient’s depression component score is 3, it implies that their depression level can be considered equivalent to that of depression of a patient whose symptom levels are all moderate (i.e., 3), suggesting that their depression is generally moderate. By Propositions 3 and 4, the means and the standard deviations of depression component are determined by weight parameter estimates within the range of its indicators’ original scales (e.g., [0, 6]) as well, which can also be interpreted in relation to those scales. For instance, if the mean of depression component scores turns out to be 5, it means that the average depression level of patients in the sample can be considered equivalent to the depression level of a patient whose symptom levels are all severe, or that the patients’ depression is severe on average. Also, if the standard deviation of depression component scores turns out to be 1, it implies that the depression severity levels of patients in the sample were one-unit lower or higher than the moderate level on average.

By Proposition 5, it is guaranteed that once a set of depression component scores is obtained with a set of weight estimates, any other set of weight estimates does not exist that makes the same score set of depression component while satisfying the constraint in (8). As these weight estimates are always non-negative and summed up to one, they can be interpreted as the indicators’ contribution ‘rates’ of forming the convex component. For example, suppose that the weight estimates for z$_{\text {1}}$, z$_{\text {2}}$ and z$_{\text {3}}$ are .41, .24, and .35, respectively. It indicates that when the severity level of depression component increases by one unit due to a one-unit increase in all the three symptom-related indicators, the contribution rates of z$_{\text {1}}$, z$_{\text {2}}$ and z$_{\text {3}}$ to the one-unit increase of depression severity are 41%, 24%, and 35%, respectively. Such interpretation was not applicable to weight of standardized components, as their values can be negative and not necessarily summed up to one. Note that this proposition is satisfied only if a linearly independent set of indicators’ scores is given as a dataset. A set of indicators’ scores being linearly independent means that a score vector of an indicator cannot be expressed as a linear combination of score vectors of the other indicators, which further implies that sample covariance matrix of the indicators is positive definite.

By Proposition 6, the path coefficient of a convex component on an outcome variable can be interpreted as an aggregate effect of the indicators of the convex component on the outcome variable, given that the structural model holds. For example, let’s consider a situation where a path coefficient of a depression component on employment earnings for the year of depression reported is identified -$5000 (e.g., Dobson et al., 2021). This would suggest that a one unit increase across all depression symptoms, such as a shift in all depression symptom levels from mild to moderate, would be associated with a $5000 loss for the individual experiencing depression. Such an interpretation was not feasible for path coefficients of standardized components.

## Convex GSCA

### Model Specification

Convex GSCA (GSCA$$_{cvx})$$ introduces a convex component with original indicators into the GSCA model. The GSCA$$_{cvx}$$ model also consists of three sub-models: weighted relation, component measurement, and structural models(Hwang and Takane, 2004, 2014). Let $${\varvec{\upgamma }} =$$ [$${{\upgamma }} _{\text {1}}$$, $${{\upgamma }}_{\text {2}}$$, $$\cdots $$, $${{\upgamma }}_{P}$$]$$^\prime $$ denote a *P* by 1 random vector of components. Each component ($$\upgamma _{p})$$ can be either a convex or standardized component. If a block of indicators (**z**$$_{p})$$ has the same measurement unit within the block, $$\upgamma _{p}$$ is defined as a convex component as expressed in (8). Otherwise, $${\varvec{\upgamma }}_{p}$$ is defined as a standardized component, whose indicators (**z**$$_{p})$$ are also assumed to be standardized such that $${\uptau }_{p} =$$ 0, $$\upphi _{p} =$$ 1, $${\varvec{\upmu }}_{p}={{\textbf {0}}}_{Jp \times 1}$$, and *vecdiag* ($$\varvec{\Sigma }_{p}) =$$ **1**$$_{Jp}$$. Let **W** denote a *J* by *P* matrix consisting of component weights assigned to **z**. Let **C** denote a *P* by *J* matrix of loadings relating $${\varvec{\upgamma }}$$ to **z**. Let **B** denote a *P* by *P* matrix of path coefficients relating $$\upgamma $$ to each other. Let $${\textbf {c}}_0$$ and $${\textbf {b}}_0$$ denote the column vectors of intercepts for the component measurement and structural models, respectively. The three sub-models of GSCA$$_{cvx}$$ are expressed as follows.

9 $$\begin{aligned} {\varvec{\upgamma }}\equiv &  {{\textbf {W}}}'{{\textbf {z}}} \,(\text {weighted relation model}) \end{aligned}$$

10 $$\begin{aligned} {{\textbf {z}}}= &  {{\textbf {c}}}_{\text {0}} + {{\textbf {C}}}' {\varvec{\upgamma }} + {\varvec{\upxi }} \,(\text {component measurement model}) \end{aligned}$$

11 $$\begin{aligned} {\varvec{\upgamma }}= &  {{\textbf {b}}}_{\text {0}} + {{\textbf {B}}}' {\varvec{\upgamma }} + {\varvec{\upzeta }} \,(\text {structural model}). \end{aligned}$$

In GSCA$_{cvx}$, the weighted relation model (9) shows that each component is defined as a weighted sum of standardized or unstandardized indicators. As GSCA$_{cvx}$ may involve unstandardized variables, intercept terms (${{\textbf {c}}_0}$ and ${{\textbf {b}}}_0$) are newly included into the component measurement and structural model (10) and (11). Each model parameter in (10) and (11)—intercepts, loadings, and path coefficients—can be interpreted in the same manner as the intercepts and regression coefficients in linear regression model with unstandardized variables. The three sub-models are combined into the following equation,

12 $$\begin{aligned} &  [\textbf{z};\varvec{\upgamma }]=[{{{\textbf {c}}}}_{\text {0}};{{{\textbf {b}}}}_{\text {0}}]+[\textbf{C},\textbf{B}]'\varvec{\upgamma }+\left[ {\varvec{\upxi };\varvec{\upzeta }} \right] \nonumber \\ &  {\leftrightarrow }\, [{{{\textbf {I}}}}_{J},\textbf{W}]'\textbf{z}=[{{{\textbf {c}}}}_{\text {0}}; {{{\textbf {b}}}}_{\text {0}}]+[\textbf{C},\textbf{B}]'\textbf{W}'\textbf{z}+\left[ {\varvec{\upxi };\varvec{\upzeta }} \right] \nonumber \\ &  {\leftrightarrow }\, {{{\textbf {V}}}'\textbf{z}=}\, {{{\textbf {a}}}}_{\text {0}} +\textbf{A}'\textbf{W}'\textbf{z}+\textbf{e}, \end{aligned}$$

where ${{\textbf {a}}}_0$ $\equiv$ [${{\textbf {c}}}_0$; ${{\textbf {b}}}_0$], **V** $\equiv$ [**I**$_{J}$, **W**], **A** $\equiv$ [**C**, **B**], and **e** $\equiv$ [${\varvec{\upxi }}$; ${\varvec{\upzeta }}$]. The equation (12) is called the GSCA$_{cvx}$ model. If every indicator and component is standardized, the GSCA$_{cvx}$ model (12) becomes identical to the GSCA$_{std}$ model (4)

### Parameter Estimation

Let ${\varvec{\upsigma }}_{p}$ denote a $J_{p}$ by 1 vector of standard deviations (SD) of **z**$_{p}$. If the *p*th component is defined as standardized ones, ${\varvec{\upsigma }}_{p}$ is equivalent to **1**$_{Jp}$. Let **O**$_{\text {z}}$ denote a *J* by *J* diagonal matrix whose *j*th element is $J_{p}^{-1}$ **1**$_{Jp}$ ${\prime }$ ${\varvec{\upsigma }}_{p}$ if the *j*th indicator in the *p*th block is a dependent variable and zero otherwise. Let **O**$_{{\upgamma }}$ denote a *P* by *P* diagonal matrix whose *p*th element is $J_{p}^{-1}$ **1**$_{Jp}$ ${\prime }$ ${\varvec{\upsigma }}_{p}$ if the *p*th component is a dependent variable and zero otherwise. Let **O** $\equiv$ *blkdiag*(**O**$_{\text {z}}$, **O**$_{{\upgamma }})$. GSCA$_{cvx}$ estimates parameters by minimizing the following objective function

13 $$\begin{aligned} &  f_{cvx}(\textbf{W,A},{{{\textbf {a}}}}_{\text {0}})\nonumber \\ &  \quad =tr(\textbf{O}E (\textbf{ee}')\textbf{O})\nonumber \\ &  \quad =E(SS(([\textbf{z};\varvec{\upgamma }]^\prime -({{{\textbf {a}}}}_{\text {0}}'+\textbf{z}'\textbf{WA}))\textbf{O})). \end{aligned}$$

subject to ${{{\textbf {w}}}}_{p}^\prime$ ${\varvec{\Sigma }}_{p}^{\prime }{{{\textbf {w}}}}_{p}$ $=$ 1 or **1**$_{Jp}\,^{\prime }{{{\textbf {w}}}}_{p} =$ 1 ($p =$ 1, 2, $\cdots$, *P*). The objective function (13) shows that components in GSCA$_{cvx}$ are constructed such that they can minimize the “weighted” sum of error variances for all dependent variables under the constraints. Specifically, the objective function (13) penalizes each prediction error for dependent variables differentially by dividing it by the average SD of the corresponding block of indicators. This prevents prediction errors for a block of indicators with large variances from dominating the estimation of parameters.

**Fig. 1:**
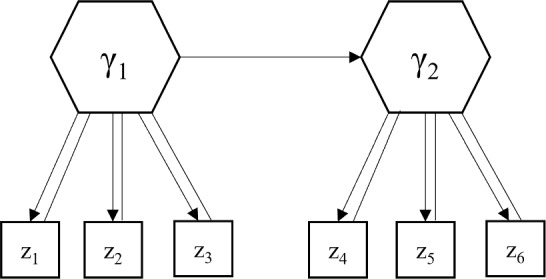
An illustrative GSCA$_{cvx}$ model. Hexagons represent components, squares denote indicators, straight lines indicate weights, single-headed arrows denote loadings and path coefficients. All intercepts and error terms are omitted to make the figure concise.

To help understand the role of **O** in (13), we illustrate how **O** is determined based on the standard deviations of indicators. This will also explain the characteristic of the objective function described above. Figure 1 presents an illustrative GSCA$$_{cvx}$$ model involving two convex components ($$\upgamma _{\text {1}}$$ and $$\upgamma _{\text {2}})$$, each measured by three indicators that share the same scale, while the scales of two indicator blocks differ. Let us assume that $${\varvec{\upsigma }}_{\text {1}} =$$ [1; 2; 3] and $${\varvec{\upsigma }}_{\text {2}} =$$ [100; 200; 300], indicating that the differences in the overall magnitude of indicators’ variances between the two blocks arises from the difference in scale. In this case, without **O** in (13) (i.e., **O** $$=$$ **I**), the value of (13) would predominantly rely on the error variances for **z**$$_{\text {2}}$$ and $$\upgamma _{\text {2}}$$, implying that the error variances for **z**$$_{\text {1}}$$ would be rarely considered in parameter estimation due to their scale. However, GSCA$$_{cvx}$$ determines **O**$$\, =\, $$ *blkdiag*(**O**$$_{\text {z}}$$, **O**$$_{{\upgamma }})$$, where **O**$$_{\text {z}} =$$ *blkdiag*(2, 2, 2, 200, 200, 200)$$^{-1}$$ and **O**$$_{{\upgamma }}=$$ *blkdiag*(0, 200$$^{-1})$$, and then uses it to penalize the error variances for **z**$$_{\text {2}}$$ and $$\upgamma _{\text {2}}$$ to adjust their effects on (13). For instance, given **A** $$=$$ **0** and **a**$$_{\text {0}} = E$$([**z**; $${\varvec{\upgamma }}$$]), there are substantial differences in error variances between **z**$$_{\text {1}}$$ and **z**$$_{\text {2}}$$ (i.e., [1$$^{\text {2}}$$; 2$$^{\text {2}}$$; 3$$^{\text {2}}$$] for **z**$$_{\text {1}}$$ and [100$$^{\text {2}}$$; 200$$^{\text {2}}$$; 300$$^{\text {2}}$$] for **z**$$_{\text {2}})$$, but their error variances contribute equally to the value of (13) (i.e., (1$$^{\text {2}}+$$ 2$$^{\text {2}} +$$ 3$$^{\text {2}})$$/2$$^{\text {2}}=$$ [100$$^{\text {2}}$$; 200$$^{\text {2}}$$; 300$$^{\text {2}}$$]/200$$^{\text {2}})$$. This suggests that introducing **O** into (13) enables GSCA$$_{cvx}$$ to consider prediction errors for both **z**$$_{\text {1}}$$ and **z**$$_{\text {2}}$$ during the parameter estimation process.

Conversely, as illustrated above, the objective function (13) does not impose different penalties on indicators within the same block to take into account potential differences in their variances. Furthermore, the objective function (13) is *partially* *scale-invariant*, which means that the minimum value of (13) does not vary with a linear change of measurement scales of each block of indicators that share the same scale (e.g., a scale range from 1–10 to 0–100), leading to the same weight estimates. This property is distinct from a property of (*full)* *scale invariant* (Swaminathan and Algina, 1978) in that changing the measurement scales of each indicator differentially (e.g., standardization) is not considered. The proof for the property is provided in Appendix 3.

As the minimum point of (13) cannot be found in closed form, we developed an ALS algorithm for iteratively finding its minimum point. A detailed description of the ALS algorithm is provided in Appendix 4. Note that we do not constrain the weights for convex components to be non-negative in (13) to make the method more flexible. In some cases, researchers may wish to examine which indicators contribute to forming a component in the opposite direction to the other indicators and may be excluded during model re-specification. The negative weight of an indicator for a convex component may signify that the indicator is not suitable to form the component along with other indicators. As discussed in Appendix 4, the ALS algorithm allows for the imposition of the additional non-negativity constraints on weights, forcing the weights to be always positive.

### Model Evaluation Indexes

GSCA$_{std}$ provides four overall goodness-of-fit measures, including FIT, AFIT, GFI, and SRMR, and one overall cross-validation index, out-of-bag prediction error (OPE). The FIT indicates the average explained variance of all variables in the model, whereas the AFIT is an adjusted version of FIT that takes into account the number of model parameters and sample size (Hwang & Takane, 2014, pp. 26–29). The GFI and SRMR evaluate the discrepancy between the sample and implied covariance matrices(Cho et al., 2020). The OPE aims to measure the average out-of-sample prediction error of the model for all variables via a bootstrapping-based cross validation and can be used for comparing models in terms of predictive generalizability(Cho et al., 2019). Whereas the GFI and SRMR can be used for GSCA$_{cvx}$ without modification, the FIT, AFIT, and OPE need to be modified for GSCA$_{cvx}$ because these measures were developed only for the condition where all variables are standardized. We revised FIT and OPE such that they can be applied for the GSCA$_{cvx}$ model with both standardized and unstandardized variables, taking into account the variances of dependent variables only.

We propose a modified version of FIT, termed FIT for unstandardized dependent variables (FIT$^{\text {UD}})$, as follows.

14 $$\begin{aligned} {\textrm{FIT}}^{\textrm{UD}}=1-\frac{SS(([{{\textbf {D}}},{{\textbf {D}}\hat{{{{\textbf {W}}}}}}]-({{\textbf {1}}}_{N} {\hat{{{\textbf {a}}}}}_{0} '+{{\textbf {D}}\hat{{\textbf {W}}}\hat{{\textbf {A}}}})){\hat{{{\textbf {O}}}}})}{SS(([{{\textbf {D}}},{{\textbf {D}}\hat{{\textbf {W}}}}]-{{\textbf {1}}}_{N} {\varvec{\hat{{\upmu }}}}'[{{\textbf {I}}}_{J},{\hat{{{\textbf {W}}}}}]){\hat{{{\textbf {O}}}}})}. \end{aligned}$$

The FIT$^{\text {UD}}$ indicates the proportion of the explained variance of all dependent variables (including dependent convex components) to their weighted total variance. If every component and indicator is standardized, FIT$^{\text {UD}} = \frac{T}{T_{Y} }$FIT, where $T \equiv P + J$ and $T_{\text {Y}}$ is the total number of dependent variables in the model. Also, we provide the following two local fit measures of FIT$^{\text {UD}}$

15 $$\begin{aligned} {\textrm{FIT}}_{\textrm{M}}^{\textrm{UD}}= &  1-\frac{SS(({{\textbf {D}}}-({{\textbf {1}}}_{N} {\hat{{{\textbf {c}}}}}_{0} '+{{\textbf {D}}\hat{{\textbf {W}}}\hat{{\textbf {C}}}})){\hat{{{\textbf {O}}}}}_{\textrm{z}} )}{SS(({{\textbf {D}}}-{{\textbf {1}}}_{N} {\varvec{\hat{{\upmu }}}}'){\hat{{{\textbf {O}}}}}_{\textrm{z}} )}, \end{aligned}$$

16 $$\begin{aligned} {\textrm{FIT}}_{\textrm{S}}^{\textrm{UD}}= &  1-\frac{SS(({{\textbf {D}}\hat{{\textbf {W}}}}-({{\textbf {1}}}_{N} {\hat{{{\textbf {b}}}}}_{0} '+{{\textbf {D}}\hat{{\textbf {W}}}\hat{{\textbf {B}}}})){\hat{{\textbf {{O}}}}}_{\upgamma } )}{SS(({{\textbf {D}}\hat{{\textbf {W}}}}-{{\textbf {1}}}_{N} {\varvec{\hat{{\upmu }}}}'{\hat{{{\textbf {W}}}}}){\hat{{{\textbf {O}}}}}_{\upgamma } )}, \end{aligned}$$

where ${\hat{{\textbf {O}}}}_{\text {z}}$ and ${\hat{{\textbf {O}}}}_{{\upgamma }}$ are sample analogies of ${{{\textbf {O}}}}_{\text {z}}$ and ${{{\textbf {O}}}}_{{\upgamma }}$. We refer to "local fit" as the goodness-of-fit of GSCA’s sub-models. The FIT$_{\text {M}}^{\text {UD}}$ and FIT$_{\text {S}}^{\text {UD}}$ can be used for evaluating the component measurement and structural models, respectively. The FIT$_{\text {M}}^{\text {UD}}$ indicates the proportion of the explained variance of all dependent indicators to their weighted total variance, whereas the FIT$_{\text {S}}^{\text {UD}}$ indicates the proportion of the explained variance of all dependent (convex) components to their weighted total variance.

Moreover, we propose a revised version of OPE, termed OPE for dependent variables (OPE$^{\text {UD}})$, to evaluate the predictive generalizability of models involving convex components, as follows.

17 $$\begin{aligned} {\textrm{OPE}}^{\textrm{UD}}=\frac{1}{K}\sum \limits _{k=1}^K {\frac{SS(([{{\textbf {D}}}_{k}^{*},{{\textbf {D}}}_{k}^{*} {\hat{{\textbf {{W}}}}}_{k} ]-({{\textbf {1}}}_{N_{k} } {\hat{{\textbf {{a}}}}}_{0} '+{{\textbf {D}}}_{k}^{*} {\hat{{\textbf {{W}}}}}_{k} {\hat{{\textbf {{A}}}}}_{k} )){\hat{{\textbf {{O}}}}}_{k} )}{SS(([{{\textbf {D}}}_{k}^{*},{{\textbf {D}}}_{k}^{*} {\hat{{{\textbf {W}}}}}_{k} ]-{{\textbf {1}}}_{N_{k} } {\hat{\varvec{{\upmu }}}}_{k} '[{{\textbf {I}}}_{J},{\hat{{{\textbf {W}}}}}_{k} ]){\hat{{\textbf {{O}}}}}_{k} )}}, \end{aligned}$$

where ${\widehat{{\textbf {W}}}}_{k}$, ${\widehat{{\textbf {A}}}}_{k}$, ${\hat{{{\textbf {a}}}}}_{k}$, and ${\varvec{{\hat{\upmu }}}}_{k}$ are the parameter estimates obtained from the *k*th bootstrap sample ($k =$ 1,2, $\cdots$, *K*), ${\hat{{\textbf {O}}}}_{k}$ is the penalty term that rescales prediction errors for all dependent variables in the *k*th bootstrap sample, ${{{\textbf {D}}}}_{k}^{*}$ is the *k*th test sample consisting of observations that are not included in the *k*th bootstrap sample, and $N_{k}$ is the number of observations in the *k*th test sample. As shown in (17), the bootstrap sampling procedure generates pairs of mutually exclusive samples (bootstrap and test samples), over which a specified GSCA model is cross-validated (for a detailed description of OPE’s computation, refer to Cho et al., 2019). The OPE$^{\text {UD}}$ represents the weighted average out-of-sample prediction error of the model for dependent variables. The value of the OPE$^{\text {UD}}$ ranges from 0 to infinity, where 0 means that a specified model perfectly predicts every dependent variable, and a value over 1 indicates that the prediction accuracy of a specified model is worse than that of the null model, where all dependent variables are predicted by their sample means. Again, when every variable is standardized, OPE$^{\text {UD}} = \frac{T}{T_{\textrm{Y}} }\textrm{OPE}-\frac{(T-T_{\textrm{Y}} )}{T_{\textrm{Y}} }$. In addition, we provide the following two local cross-validation indexes of OPE$^{\text {UD}}$

18 $$\begin{aligned} {\textrm{OPE}}_{\textrm{M}}^{\textrm{UD}}= &  \frac{1}{K}\sum \limits _{k=1}^K {\frac{SS(({{\textbf {D}}}_{k}^{*} -({{\textbf {1}}}_{N_{k} } {\hat{{{\textbf {c}}}}}_{0,k} '+{{\textbf {D}}}_{k}^{*} {\hat{{{\textbf {W}}}}}_{k} {\hat{{\textbf {{C}}}}}_{k} )){\hat{{\textbf {{O}}}}}_{{\textrm{z}},k} )}{SS(({{\textbf {D}}}_{k}^{*} -{{\textbf {1}}}_{N_{k} } {\hat{\varvec{{\upmu }}}}_{k} '){\hat{{{\textbf {O}}}}}_{\mathrm{\textrm{z}},k} )}}, \end{aligned}$$

19 $$\begin{aligned} {\textrm{OPE}}_{\textrm{S}}^{\textrm{UD}}= &  \frac{1}{K}\sum \limits _{k=1}^K {\frac{SS(({{\textbf {D}}}_{k}^{*} {\hat{{\textbf {{W}}}}}_{k} -({{\textbf {1}}}_{N_{k} } {\hat{{\textbf {{b}}}}}_{0,k} '+{{\textbf {D}}}_{k}^{*} {\hat{{{\textbf {W}}}}}_{k} {\hat{{{\textbf {B}}}}}_{k} )){\hat{{\textbf {{O}}}}}_{\upgamma ,k} )}{SS(({{\textbf {D}}}_{k}^{*} {\hat{{\textbf {{W}}}}}_{k} -{{\textbf {1}}}_{N_{k} } {\hat{\varvec{{\upmu }}}}_{k} '{\hat{{\textbf {{W}}}}}_{k} ){\hat{{\textbf {{O}}}}}_{\upgamma ,k} )}}, \end{aligned}$$

where ${\hat{{\textbf {O}}}}_{{\textrm{z}},k}$ and ${\hat{{\textbf {O}}}}_{\upgamma ,k}$ are the penalty terms that rescale prediction errors for dependent indicators and components, respectively, in the *k*th bootstrap sample. The OPE$_{\text {M}}^{\text {UD}}$ and OPE$_{\text {S}}^{\text {UD}}$ can be used for evaluating the predictive generalizability of the component measurement and structural models, respectively.

## Simulated Data Analysis

**Fig. 2:**
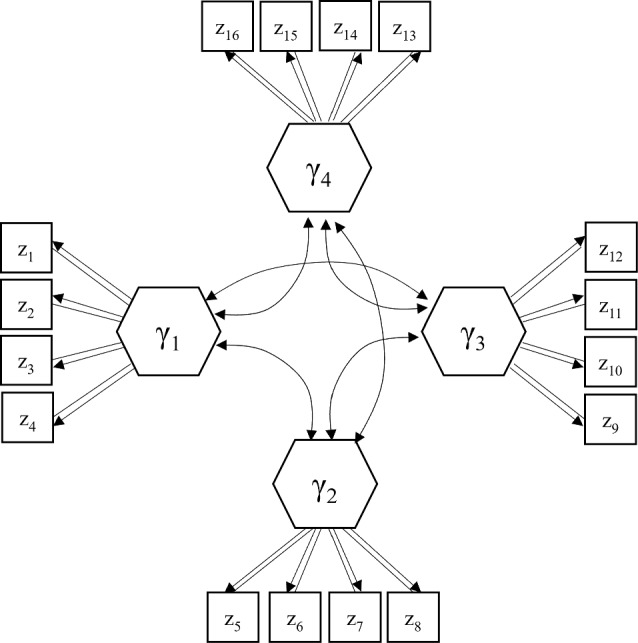
The population GSCA$_{cvx}$ model used in the simulation study. Double-headed arrows represent correlations. All intercepts and error terms are omitted to make the figure concise.

We conduct a simulation study to examine the parameter recovery of the proposed method. Figure 2 depicts the population GSCA$_{cvx}$ model used in our simulation study. The population model involves four convex components, each of which is measured by four composite indicators. Indicators per block had different mean vectors: the mean vectors of indicators are [6, 5, 4, 3] for $\upgamma _{\text {1}}$, [5.5, 4.5, 3.5, 2.5] for $\upgamma _{\text {2}}$, [5, 4, 3, 2] for $\upgamma _{\text {3}}$, and [4.5, 3.5, 2.5, 1.5] for $\upgamma _{\text {4}}$, respectively.

We manipulate four experimental factors: the variances of indicators, correlations between indicators per component, distribution of indicators, and correlations among components. We consider the variances of indicators because this is a unique piece of information for the proposed method to use for creating components as compared to GSCA$_{std}$. The other three factors have been frequently considered in testing the performance of GSCA (e.g., Cho et al., 2022; Cho & Choi, 2020; Hwang, Malhotra, et al., 2010b). Specifically, we consider three levels of the variances of indicators per component: [1, 1, 1, 1], [1 2, 3, 4], and [1, 4, 9, 16]. We take into account three correlation matrices of indicators per component, which are provided in Table 1 (Cho and Choi, 2020). We consider two distributions of indicators: normal and non-normal. The normal distribution has a skewness of 0 and a kurtosis of 3, whereas the non-normal distribution has a skewness of 1.25 and a kurtosis of 3.75 as in Hwang et al. (2010a). Lastly, we consider three levels of correlations among components (0, .2, and .4) as in Cho et al. (2022). In total, we consider 54 population GSCA models with convex components (3 levels of indicators’ variances $\times$ 2 types of indicators’ distribution $\times$ 3 levels of indicators’ correlations $\times$ 3 levels of components’ correlations).

**Table 1:** Three conditions of the correlation patterns of four indicators per component in the simulation study.

	Condition 1				Condition 2				Condition 3			
	z$_{\text {1}}$	z$_{\text {2}}$	z$_{\text {3}}$	z$_{\text {4}}$	z$_{\text {1}}$	z$_{\text {2}}$	z$_{\text {3}}$	z$_{\text {4}}$	z$_{\text {1}}$	z$_{\text {2}}$	z$_{\text {3}}$	z$_{\text {4}}$
z$_{\text {1}}$	1				1				1			
z$_{\text {2}}$	.24	1			.50	1			.49	1		
z$_{\text {3}}$	.24	.20	1		.43	.47	1		.56	.74	1	
z$_{\text {4}}$	.17	.21	.13	1	.30	.23	.45	1	.66	.48	.69	1

Per population model, we consider five sample sizes ($N =$ 100, 200, 400, 800, and 1500), for each of which 1000 samples are randomly generated from the multivariate distribution with the population mean vector and covariance matrix of indicators. The procedure of deriving the population covariance matrix of indicators from the prescribed parameter values of a population GSCA$_{cvx}$ model is explained in Appendix 5. We apply GSCA$_{cvx}$^1^ to each sample and obtain parameter estimates.

As parameter recovery measures, we empirically compute the absolute bias and root mean squared error (RMSE) of each parameter estimator. These measures are defined as

20 $$\begin{aligned} \hbox {Absolute\,bias}= &  \left| {{\textrm{E}}(\hat{{\uptheta }})-\uptheta } \right| \approx \left| {\frac{1}{\text{1000 }}(\sum \limits _{i=1}^{1000} {\hat{{\uptheta }}_{i} )-\uptheta } } \right| , \end{aligned}$$

21 $$\begin{aligned} \textrm{RMSE}= &  \sqrt{{\textrm{E}}(\hat{{\uptheta }}-\uptheta )^{2}} \approx \sqrt{\frac{1}{1000}\sum \limits _{i=1}^{1000} {(\hat{{\uptheta }}_{i} -\uptheta )^{2}} }, \end{aligned}$$

where $\uptheta$ is the value of each parameter, $\hat{{\uptheta }}$ is the estimator of $\uptheta$, and $\hat{{\uptheta }}_{i}$ is the estimate of $\uptheta$ obtained from the *i*th sample. We focus here on reporting the average absolute bias and RMSE values of the estimators of weights, loadings, intercepts, component means, and component variances over the population models per sample size, as the sample size is the only factor that substantially influences the absolute bias and RMSE values of the estimators. The results for each population model are provided in Supplementary Material.

Table 2 shows the average absolute bias and RMSE values of the estimators per sample size. In all sample sizes, the absolute biases of the weight, loading, and component mean estimators are small and close to zero on average. For example, when $N =$ 100, the average absolute biases of the weight, loading, and component mean estimators are .002, .022, and .008, respectively. They continue to decrease and approach zero when the sample size increases. The average RMSE values of the same estimators show a similar pattern. When $N =$ 100, the average RMSE values are around .047, .134, and .216, respectively, and becomes close to zero as the sample size increases. The average absolute bias and RMSE values of the intercept and component variance estimators are relatively large, compared to those of the other parameter estimators in the same condition. For instance, when $N =$ 100, the average absolute biases of the intercept and component variance estimators are .107 and .178, respectively, and their average RMSE values are .668 and .859, respectively. However, both of them also decrease with the sample size and become close to zero. Taken together, GSCA$_{cvx}$ estimators are empirically unbiased on average, improving their parameter recovery as the sample size increases.

**Table 2:** The average absolute bias and RMSE values of the estimators of weights, loadings, intercepts, component means, and component variances per sample size.

	Absolute Bias					RMSE				
N	Weights	Loadings	Intercepts	Component Means	Component Variances	Weights	Loadings	Intercepts	Component Means	Component Variances
100	0.002	0.022	0.107	0.008	0.178	0.047	0.134	0.668	0.216	0.859
200	0.001	0.011	0.052	0.005	0.078	0.030	0.092	0.460	0.146	0.515
400	0.001	0.005	0.026	0.003	0.037	0.021	0.064	0.319	0.102	0.346
800	0.000	0.003	0.013	0.002	0.018	0.014	0.045	0.223	0.072	0.240
1500	0.000	0.002	0.008	0.002	0.010	0.010	0.033	0.162	0.052	0.174

## Illustration with Empirical Data

To illustrate its empirical utility, we apply GSCA$_{cvx}$ to American customer satisfaction index (ACSI) data. The ACSI model (Fornell et al., 1996) is built on the established theories and has been used to produce index scores for customer satisfaction in the United States since 1994. The present ACSI data are comprised of 774 customers’ responses for fourteen items: z$_{\text {1}} =$ expectation for overall quality, z$_{\text {2}} =$expectation for reliability, z$_{\text {3}} =$ expectation for customization, z$_{\text {4}} =$ overall quality, z$_{\text {5}} =$ reliability, z$_{\text {6}} =$ customization, z$_{\text {7}} =$ price given quality, z$_{\text {8}}$ $=$ quality given price, z$_{\text {9}} =$ perceived overall satisfaction, z$_{\text {10}} =$ fulfilment of expectations, z$_{\text {11}} =$ distance to the ideal, z$_{\text {12}} =$ complaint behavior, z$_{\text {13}} =$ repurchase intention, z$_{\text {14}} =$ price tolerance. Twelve of the items (z$_{\text {1}}$,z$_{\text {2}}$, z$_{\text {3}}$, z$_{\text {4}}$, z$_{\text {5}}$, z$_{\text {6}}$, z$_{\text {7}}$, z$_{\text {8}}$, z$_{\text {9}}$, z$_{\text {10}}$, z$_{\text {11}}$, and z$_{\text {13}})$ are measured on a 10-point Likert scale (e.g., 1 $=$ “very negative” and 10 $=$ “very positive”). Within the interval [1, 5], a smaller point reflects a stronger negative response, whereas within the interval [6, 10], a larger point indicates a stronger positive response. On the other hand, z$_{\text {12}}$ is a binary variable (1 $=$ formally complained and 0 $=$ otherwise) and z$_{\text {14}}$ is a composite of two price tolerance measures in different metrics, which is expressed as a percentage ranging from 0 to 50 (the higher, the more tolerant). The means, covariances, minimums, and maximums of the items are provided in Table 3. Refer to Fornell et al. (1996) for more detailed information on the items.

**Table 3:** Sample covariances (in upper triangular), correlations (in lower triangular), variances (in diagonal), means, minimums, and maximums of the fourteen indicators in the ACSI example.

	z$_{\text {1}}$	z$_{\text {2}}$	z$_{\text {3}}$	z$_{\text {4}}$	z$_{\text {5}}$	z$_{\text {6}}$	z$_{\text {7}}$	z$_{\text {8}}$	z$_{\text {9}}$	z$_{\text {10}}$	z$_{\text {11}}$	z$_{\text {12}}$	z$_{\text {13}}$	z$_{\text {14}}$
z$_{\text {1}}$	5.81	3.61	2.71	2.92	2.91	2.10	1.96	2.79	3.01	2.73	3.23	–.14	2.62	11.78
z$_{\text {2}}$	.65	5.38	2.88	2.69	2.92	2.36	1.48	2.63	2.79	2.30	2.64	–.13	2.05	7.65
z$_{\text {3}}$	.43	.47	6.90	2.03	2.15	2.56	1.49	2.18	2.16	1.96	2.41	–.12	1.74	7.77
z$_{\text {4}}$	.53	.50	.33	5.31	4.77	3.76	2.52	4.15	4.87	4.35	4.42	–.32	3.94	16.86
z$_{\text {5}}$	.49	.51	.33	.84	6.11	3.98	2.48	4.37	5.23	4.80	5.01	–.36	4.29	17.20
z$_{\text {6}}$	.33	.39	.37	.62	.61	6.93	2.46	3.65	3.95	3.67	3.75	–.23	3.18	13.58
z$_{\text {7}}$	.31	.25	.22	.42	.39	.36	6.67	3.43	3.05	2.88	2.78	–.14	2.42	11.31
z$_{\text {8}}$	.47	.46	.33	.72	.71	.56	.53	6.18	4.74	4.42	4.70	–.29	3.60	16.60
z$_{\text {9}}$	.50	.48	.33	.85	.85	.60	.47	.77	6.19	5.06	5.09	–.35	4.55	19.55
z$_{\text {10}}$	.45	.40	.30	.75	.78	.56	.44	.71	.81	6.27	4.96	–.29	3.98	16.71
z$_{\text {11}}$	.51	.43	.35	.73	.77	.54	.41	.72	.78	.75	6.93	–.32	4.74	20.79
z$_{\text {12}}$	–.17	–.17	–.13	–.40	–.42	–.25	–.16	–.34	–.41	–.34	–.36	.12	–.33	-1.55
z$_{\text {13}}$	.37	.30	.22	.58	.58	.41	.32	.49	.62	.54	.61	–.33	8.79	35.21
z$_{\text {14}}$	.31	.21	.19	.47	.45	.33	.28	.43	.51	.43	.51	–.29	.76	241.11
Mean	7.34	7.75	6.67	7.66	7.59	7.39	5.96	7.12	7.59	6.82	6.76	.14	7.73	31.82
Min	1	1	1	1	1	1	1	1	1	1	1	0	1	0
Max	10	10	10	10	10	10	10	10	10	10	10	1	10	50

**Fig. 3:**
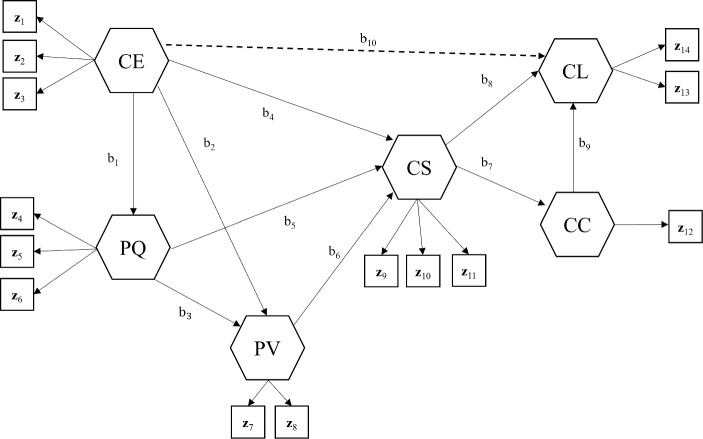
The ACSI model. The dashed line labeled b$_{\text {10}}$ signifies an incorrectly specified path coefficient. All weights and error terms are omitted to make the figure concise. CE $=$ customer expectations, PQ $=$ perceived quality, PV $=$ perceived value, CS $=$ customer satisfaction, CC $=$ customer complaints, CL $=$ customer loyalty.

Figure 3 depicts the relationships among the six components and their indicators. The 14 items are used as composite indicators of the following six components: $\upgamma _{\text {1}} =$ customer expectations (CE), $\upgamma _{\text {2}} =$ perceived quality (PQ), $\upgamma _{\text {3}} =$ perceived value (PV), $\upgamma _{\text {4}} =$ customer satisfaction (CS), $\upgamma _{\text {5}} =$ customer complaints (CC), and $\upgamma _{\text {6}}=$ customer loyalty (CL). We represent all the constructs by convex components with unstandardized indicators except for the customer loyalty. As two indicators (z$_{\text {13}}$ and z$_{\text {14}})$ for customer loyalty are not measured on the same scale, we set this component as a standardized one with the indicators standardized.

We use 4000 bootstrap samples for computing the standard error and 95% confidence interval of each parameter estimate. For comparison, we also apply GSCA$_{std}$ to the same data and compute unstandardized weight estimates and unstandardized component scores based on the procedure discussed in Sect. 1. As customer satisfaction is the focal component in the ACSI model, we concentrate on interpreting the scores of customer satisfaction, its statistics, and the relevant model parameters.

The model fitted by GSCA$_{cvx}$ shows FIT$^{\text {UD}} =$.714, indicating that the ACSI model accounts for 71.4% of the weighted total variance of all dependent variables in the model. It also provides GFI $=$ .987 and SRMR $=$ .022, pointing to an acceptable level of model fit(Cho et al., 2020). In addition, it provides that FIT$_{\text {M}}^{\text {UD}}$ $=$ .802 and FIT$_{\text {S}}^{\text {UD}} =$ .438. This indicates that the component measurement model explains 80.2% of the weighted total variance of all dependent indicators, whereas the structural model explains 43.8% of the weighted total variance of all dependent components.

Table 4 provides the weight and loading estimates, and their standard errors and 95% confidence intervals obtained from GSCA$_{cvx}$, along with the intercept estimates in the measurement model. The unstandardized weight estimates obtained from GSCA$_{std}$ are also provided for comparison. Overall, all the weight and loading estimates obtained from GSCA$_{cvx}$ are large and statistically significant, indicating that all the indicators contribute to forming their components, which in turn, explain the variances of their indicators well. Among the three indicators (z$_{\text {9}}$, z$_{\text {10}}$, and z$_{\text {11}})$ for customer satisfaction, z$_{\text {9}}$ (perceived overall satisfaction) are the largest contributor (w$_{\text {9}}=$ .422, SE $=$ .015, 95% CI $=$ [.393, .454]). This indicates that when each of the three indicators equally increases, leading to an increase in customer satisfaction, the contribution rate of **z**$_{\text {9}}$ for the increase in customer satisfaction was 42.2%, which is greater than those of the two others (z$_{\text {10}} =$ 25.4% and z$_{\text {11}} =$ 32.4%). Similarly, the unstandardized weight estimate of **z**$_{\text {9}}$ obtained from GSCA$_{std}$ is the largest among the three (w$_{\text {9}}$ $=$ .188, w$_{\text {10}} =$ .107, and w$_{\text {11}}=$ .131). In contrast, it is uncertain how to interpret the unstandardized weight estimates obtained from GSCA$_{std}$.

**Table 4:** The weights, loading, and intercept estimates of the fourteen indicators in the ACSI model and their standard errors (SE) and 95% confidence intervals (CI) obtained from GSCA$_{cvx}$, along with the unstandardized weight estimates obtained from GSCA$_{std}$ ($\pmb {\hat{\textrm{W}}}_{\!uni})$.

Indicator	Component	Weights			$\pmb {\hat{\textrm{W}}}_{\!uni}$	Loadings			Intercepts($\pmb {\hat{\textrm{c}}}_{\text {0}})$
		Estimate	SE	95% CI		Estimate	SE	95% CI	
z$_{\text {1}}$	CE	.345	.013	[.320, .372]	.180	1.008	.025	[.957, 1.054]	.018
z$_{\text {2}}$		.337	.014	[.309, .366]	.188	.982	.027	[.925, 1.033]	.616
z$_{\text {3}}$		.317	.013	[.292, .343]	.128	1.011	.036	[.937, 1.077]	–.674
z$_{\text {4}}$	PQ	.387	.018	[.353, .425]	.184	.979	.013	[.953, 1.004]	.260
z$_{\text {5}}$		.342	.017	[.307, .374]	.170	1.043	.013	[1.018, 1.071]	–.303
z$_{\text {6}}$		.271	.007	[.257, .285]	.101	.976	.024	[.927, 1.021]	.012
z$_{\text {7}}$	PV	.404	.010	[.384, .423]	.154	.960	.020	[.921, .997]	–.427
z$_{\text {8}}$		.596	.010	[.577, .616]	.293	1.027	.013	[1.002, 1.051]	.289
z$_{\text {9}}$	CS	.422	.015	[.393, .454]	.188	1.004	.011	[.982, 1.025]	.433
z$_{\text {10}}$		.254	.013	[.229, .279]	.107	.965	.016	[.933, .996]	–.052
z$_{\text {11}}$		.324	.012	[.300, .348]	.131	1.022	.016	[.990, 1.053]	–.524
z$_{\text {12}}$	CL	1.000	.000	[1.000, 1.000]	2.909	1.000	.000	[1.000, 1.000]	.000
z$_{\text {13}}$	CC	.610	.015	[.579, .639]	.206	.956	.004	[.949, .963]	.000
z$_{\text {14}}$		.453	.016	[.424, .484]	.029	.920	.007	[.906, .932]	.000

Table 5 presents the path coefficient estimates and their standard errors and 95% confidence intervals obtained from GSCA$_{cvx}$. Overall, the patterns of all the path coefficient estimates are consistent with those from previous studies (e.g., Hwang & Takane, 2014, Chapter 2). For instance, perceived quality and perceived value have statistically significant influences on customer satisfaction (b$_{\text {5}} =$ .723, SE $=$ .033, 95% CI $=$ [.659, .786]; b$_{\text {6}} =$ .275, SE $=$ .035, 95% CI $=$ [.204, .344]). Customer satisfaction have statistically significant effects on customer complaints (b$_{\text {7}} =$ –.059, SE $=$ .006, 95% CI $=$ [–.072, –.047]) and customer loyalty (b$_{\text {8}} =$ .252, SE $=$ .015, 95% CI $=$ [.222, .279]). Each individual path coefficient estimate is indicative of the expected change of the dependent component for a one-unit change in indicators of a predictor component. For instance, the estimate of the path coefficient, b$_{\text {8}} =$ .252, implies that a one-unit increase in z$_{\text {9}}$ (perceived overall satisfaction), z$_{\text {10}}$ (expectation fulfillment), and z$_{\text {11}}$ (distance to the ideal) would be associated with an increase of .252 unit in customer loyalty. The $R^{\text {2}}$ value is .331 for perceived quality, .511 for perceived value, .812 for customer satisfaction, .164 for customer complaints, and .404 for customer loyalty. Also, the intercept estimates for the dependent components in the same order as above are 3.014, .793, –.501, .558, and $-$1.756.

**Table 5:** The path efficient estimates and their standard errors (SE) and 95% confidence intervals (CI) obtained from GSCA$_{cvx}$.

		Estimate	SE	95% CI
b$_{\text {1}}$	CE $\rightarrow$ PQ	.626	.037	[.551, .696]
b$_{\text {2}}$	CE $\rightarrow$ PV	.134	.039	[.058, .209]
b$_{\text {3}}$	PQ $\rightarrow$ PV	.646	.038	[.573, .721]
b$_{\text {4}}$	CE $\rightarrow$ CS	.045	.026	[–.005, .095]
b$_{\text {5}}$	PQ $\rightarrow$ CS	.723	.033	[.659, .786]
b$_{\text {6}}$	PV $\rightarrow$ CS	.275	.035	[.204, .344]
b$_{\text {7}}$	CS $\rightarrow$ CC	–.059	.006	[–.072, –.047]
b$_{\text {8}}$	CS $\rightarrow$ CL	.252	.015	[.222, .279]
b$_{\text {9}}$	CC$\rightarrow$ CL	–.267	.104	[–.471, –.064]

Table 6 presents the estimated means, standard deviations, and ranges of unstandardized component scores obtained from GSCA$_{cvx}$ and GSCA$_{std}$. As expected, the individual scores of each convex component obtained from GSCA$_{cvx}$ are within the range of their indicators’ scores. The individual scores of customer expectation, perceived quality, perceived value, and customer satisfaction all range from 1 to 10 and those of customer complaint were between 0 and 1, which are equivalent to the ranges of their indicators’ measurement scales. The mean of customer satisfaction from GSCA$_{cvx}$ is 7.125, indicating that the average satisfaction level in the sample is moderately positive or equivalent to the satisfaction level of a customer whose indicator scores are all 7.125. This mean of customer satisfaction appears to be congruent with the means of its original indicators (7.585, 6.824, and 6.760). The standard deviation of customer satisfaction is 2.353, suggesting that the scores of customer satisfaction are somewhat widely spread out from the mean. This standard deviation value also seems to conform to those of its original indicators (2.489, 2.504, and 2.632).

**Table 6:** The means, standard deviations (SD), and ranges of the unstandardized component scores estimated from GSCA$_{cvx}$ and GSCA$_{std}$. The last component (CL) is defined as a standardized component in GSCA$_{cvx}$.

	GSCA$_{cvx}$			GSCA$_{std}$		
	Mean	SD	Range	Mean	SD	Range
CE	7.265	2.014	[1.000, 10.000]	3.633	1.000	[.496, 4.961]
PQ	7.564	2.194	[1.000, 10.000]	3.444	1.000	[.455, 4.546]
PV	6.652	2.223	[1.000, 10.000]	3.008	1.000	[.448, 4.475]
CS	7.125	2.353	[1.000, 10.000]	3.037	1.000	[.425, 4.253]
CC	0.137	0.344	[0.000, 1.000]	.398	1.000	[.000, 2.909]
CL	0.000	1.000	[–2.311, .998]	2.518	1.000	[.206, 3.516]

On the contrary, unstandardized components’ scores obtained from GSCA$_{std}$ are not always within the range of their indicators’ scores. Some scores of customer expectation, perceived quality, perceived value, and customer satisfaction are smaller than 1, which is the minimum value of their indicators on the scale. Moreover, the means of unstandardized components are also far from those of their original indicators. For instance, the mean of customer satisfaction obtained from GSCA$_{std}$ is just 3.037, even though its indicators’ means are around 7 as stated above. Thus, it is questionable whether the mean of customer satisfaction obtained from GSCA$_{std}$ can be a good representation of the average level of customer satisfaction in the sample. Furthermore, all the standard deviations of unstandardized components are fixed to one, even though none of their indicators have standard deviations being around 1.

To illustrate the usage of OPE$^{\text {UD}}$ as a model comparison criterion, we additionally contemplate two misspecified models of the ACSI model, while assuming the original ACSI model as the true model (denoted by Model 1). One misspecified model (Model 2) is an under-specified one, where a path coefficient (b$_{\text {6}})$ is omitted from Model 1. The other misspecified model (Model 3) is an over-specified one that includes an additional path coefficient from customer expectation to customer loyalty in Model 1, as displayed in Fig. 3. We apply GSCA$_{cvx}$ to fit the three models to the data and compute their OPE$^{\text {UD}}$ values based on 4000 bootstrap samples. Model 1 provides the smallest OPE$^{\text {UD}}$ value (Model 1 $=$ .2883, Model 2 $=$ .2901, and Model 3 $=$ .2887), indicating that the original ACSI model has the highest predictive generalizability among the three models. The OPE$^{\text {UD}}$ value of Model 2 is larger than that of Model 1 (.2901 >.2883), suggesting that excluding a path coefficient (b$_{\text {6}})$ from Model 1 rather decreases the prediction accuracy of the model. On the other hand, the OPE$^{\text {UD}}$ value of Model 3 is larger than that of Model 1 (.2887 >.2883), indicating that specifying an additional path coefficient (b$_{\text {10}})$ to Model 1 is not helpful to improve the predictive generalizability of the model.

## Concluding Remarks

We proposed convex GSCA that can accommodate a new type of unstandardized components, named convex components. A convex component is defined as a convex combination of original indicators whose weights are all non-negative and summed up to one. Every individual score of a convex component is always within the range of its indicators’ scores and can be interpreted as a construct’s specific level of a person who has the same score for all its indicators as his/her component score. Moreover, the means and standard deviations of convex components are estimated along with other parameters through a single optimization procedure, which can also be interpreted in terms of indicators’ scales. Thus, introducing convex components to the GSCA model will enhance the practical utility of component scores and their summary statistics, for instance, in investigating individuals’ levels of a construct or comparing the average levels of a construct between groups.

We developed an alternating least squares (ALS) algorithm for estimating parameters of the convex GSCA model, which does not require standardizing blocks of indicators that have the same measurement scales within the blocks. The algorithm not only enables information on the variances of each block of indicators to be additionally utilized in parameter estimation, but also prevents indicators with small variances from influencing more heavily the construction of an unstandardized component than those with large variances. Furthermore, its objective function is partially scale-invariant, indicating that the minimum value of the objective function remains unchanged with a linear change in the measurement scale of each block of indicators, giving rise to the same weight estimates.

We evaluated the parameter recovery of the proposed method in a simulation study and further illustrated the merits of the proposed method via a real data analysis. In the simulation study, the proposed method empirically produced unbiased parameter estimates on average under nine GSCA models with convex components and its accuracy was further improved with large sample size. In the real data analysis, the patterns of the parameter estimates were consistent with those from previous studies, and the benefits of convex components were pronounced, compared to the unstandardized components obtained from the conventional ad-hoc procedure of rescaling weight estimates. Unlike these unstandardized components, convex components’ weight estimates were interpretable, all their individual scores fell within the range of indicators’ measurement scales or their scores, and their estimated means and standard deviations were congruous with those of their indicators. Therefore, we are confident to recommend that researchers employ the method when they are interested in the GSCA model with unstandardized components of original indicators.

Note that as an anonymous reviewer pointed out, researchers may still want to consider standardizing observed variables that are measured on the same scale. We recommend considering this option only if researchers are not interested in unstandardized component scores. If researchers apply GSCA to estimate the scores of unstandardized components after standardizing indicators of the same scale, an indicator with a small variance can be assigned a relatively large unstandardized weight, leading to a potentially inflated influence of the indicator on the estimation of the component scores, as shown in Sect. 1. This issue does not occur when researchers keep the original scales of indicators and apply convex GSCA with convex components.

In future research, we may consider incorporating convex components into various extensions of GSCA, which deal with more complex analyses, for instance, those of involving higher-order components (Hwang & Takane, 2014, Chapter 3), missing observations (Hwang & Takane, 2014, Chapter 3), multilevel components (Hwang et al., 2007), components with categorical indicators (Hwang and Takane, 2010), component interaction terms(Hwang et al., 2021a, 2010a), or factors (Hwang et al., 2021b). Such additional extensions will improve the usefulness of GSCA, placing components on their indicators’ scales while having their means and variances free parameters to be estimated along with others.

## Appendix 1. A Proof of Disproportional Penalty Imposition on Indicators During the Minimization of the Objective Function (5)

Let ${{{\textbf {z}}}}$ $=$ ${\varvec{\upmu }} +{\varvec{\Delta }}_{\text {z}}{{{\textbf {z}}}}_{std}$ is a random vector of original indicators, where ${\varvec{\upmu }}$, ${\varvec{\Sigma }}$, and ${\varvec{\Delta }}_{\text {z}}$ denote a column mean vector of **z**, the covariance matrix of **z**, and a diagonal matrix consisting of each indicator’s standard deviation, respectively. Let ${\varvec{\upgamma }}_{uni} \equiv$ ${{{\textbf {W}}}}_{uni}'\textbf{z}$ denote a random vector of unstandardized components, where ${{{\textbf {W}}}}_{uni}$ is a matrix of unstandardized weight parameters and *vecdiag*(${{{\textbf {W}}}}_{uni}'\varvec{\Sigma }{{{\textbf {W}}}}_{uni})$ $=$ **1**$_{P}$. Let ${{{\textbf {e}}}}_{uni}$ is a random vector of prediction errors for $[\textbf{z};{\varvec{\upgamma }}_{uni}]$. Let ${\varvec{\Delta }} \equiv$*blkdiag*(${\varvec{\Delta }}_{\text {z}}$, **I**$_{P}$) is a diagonal matrix of penalty parameters for ${{{\textbf {e}}}}_{uni}$, where *blkdiag*() is an operator to convert input matrices into a block-diagonal matrix. Here, the penalty parameters refer to the parameters that rescale prediction error for each dependent variable in the model. Let ${{{\textbf {A}}}}_{uni}$ denote a matrix of unstandardized loading and path coefficients in GSCA$_{std}$. Let ${{{\textbf {a}}}}_{\text {0,}uni}$ denote a column vector of the unstandardized intercepts in GSCA$_{std}$. When ${{{\textbf {W}}}}_{uni}$ $= {\varvec{\Delta }}_{\text {z}}^{{-1}}{{{\textbf {W}}}}_{std}$, ${{{\textbf {A}}}}_{uni}={{{\textbf {A}}}}_{std}{\varvec{\Delta }}$, and ${{{\textbf {a}}}}_{\text {0,}uni}=([\textbf{I}_{J} , {{{\textbf {W}}}}_{uni}]-{{{\textbf {W}}}}_{uni}{{{\textbf {A}}}}_{uni})^\prime$ ${\varvec{\upmu }}$, (5) is equivalent to the following objective function,

A.1 $$\begin{aligned} &  f_{uni}({{{\textbf {W}}}}_{uni},{{{\textbf {A}}}}_{uni},{{{\textbf {a}}}}_{\text {0,}uni})\nonumber \\ &  \quad =tr({\varvec{\Delta }}^{{-1}}E({{{\textbf {e}}}}_{uni}{{{\textbf {e}}}}_{uni}^{\prime }){\varvec{\Delta }}^{{-1}}) \end{aligned}$$

subject to *vecdiag*(${{{\textbf {W}}}}_{uni}'\varvec{\Sigma }{{{\textbf {W}}}}_{uni})$ $=$ **1**$_{P}$, which can be proved as follows.

A.2 $$\begin{aligned} &  f_{std}({{{\textbf {W}}}}_{std},{{{\textbf {A}}}}_{std})\nonumber \\ &  \quad =tr(E({{{\textbf {e}}}}_{std}{{{\textbf {e}}}}_{std}\,^\prime ))\nonumber \\ &  \quad =E(SS({{{\textbf {z}}}}_{std}'{{{\textbf {V}}}}_{std}-{{{{\textbf {z}}}}_{std}'\textbf{W}}_{std}{{{\textbf {A}}}}_{std}\text {)))}\nonumber \\ &  \quad =E(SS((\textbf{z}-\varvec{\upmu }\mathbf {)}{'{\varvec{\Delta }}_{\text {z}}}^{{-1}}({{{\textbf {V}}}}_{std}-{{{\textbf {W}}}}_{std}{{{\textbf {A}}}}_{std}\text {)))}\nonumber \\ &  \quad =E(SS((\textbf{z}-\varvec{\upmu }\mathbf {)}'([{\varvec{\Delta }}_{\text {z}}^{{-1}},{\varvec{\Delta }}_{\text {z}}^{{-1}}{{{\textbf {W}}}}_{std}]-{\varvec{\Delta }}_{\text {z}}^{{-1}}{{{\textbf {W}}}}_{std}{{{\textbf {A}}}}_{std}{\varvec{\Delta }}{\varvec{\Delta }}^{{-1}}\text {)))}\nonumber \\ &  \quad =E(SS((\textbf{z}-\varvec{\upmu }\mathbf {)}^\prime ([{{{\textbf {I}}}}_{J},{{{\textbf {W}}}}_{uni}\text {]}{\varvec{\Delta }}^{{-1}}-{{{\textbf {W}}}}_{uni}{{{\textbf {A}}}}_{uni}{\varvec{\Delta }}^{{-1}}\text {)))}\nonumber \\ &  \quad =E(SS((\textbf{z}-\varvec{\upmu }\mathbf {)}^\prime ({{{\textbf {V}}}}_{uni}-{{{\textbf {W}}}}_{uni}{{{\textbf {A}}}}_{uni}){\varvec{\Delta }}^{{-1}}))\nonumber \\ &  \quad =E(SS\text {((}{{{{\textbf {z}}}'\textbf{V}}}_{uni}-{(\textbf{z}'}{{{\textbf {W}}}}_{uni}{{{\textbf {A}}}}_{uni}+{{{\textbf {a}}}}_{\text {0,}uni}\,^\prime )){\varvec{\Delta }}^{{-1}}\text {)),}\nonumber \\ &  \quad =tr({\varvec{\Delta }}^{{-1}}E({{{\textbf {e}}}}_{uni}{{{\textbf {e}}}}_{uni}\,^\prime ){\varvec{\Delta }}^{{-1}})\nonumber \\ &  \quad =f_{std}({{{\textbf {W}}}}_{uni},{{{\textbf {A}}}}_{uni},{{{{\textbf {a}}}}}_{\text {0,}uni}), \end{aligned}$$

where **V**$$_{uni} \equiv $$$$\text {[}{{{\textbf {I}}}}_{J},{{{\textbf {W}}}}_{uni}\text {]}$$. The equivalence between (5) and (A.1) indicates that GSCA$$_{std}$$’s parameters are actually the standardized versions of $${{{\textbf {W}}}}_{uni}$$ and $${{{\textbf {A}}}}_{uni}$$ that are obtained by minimizing the sum of penalized error variances for the original indicators and unstandardized components. While minimizing (A.1), a relatively large penalty will be imposed on an indicator with a relatively large variance, potentially inflating the influence of an indicator with a small variance on GSCA$$_{std}$$’s parameter estimation.

## Appendix 2. Proofs of the Six Propositions that Characterize a Convex Component

Let us suppose that the *p*th component ($$\upgamma _{p})$$ is a convex component defined with $$J_{p}$$ indicators (**z**$$_{p})$$, indicating that the sum of weights assigned to the indicators is equal to one (i.e., **1**$$_{Jp}^\prime $$ **w**$$_{p}=$$ 1) and all the weights are non-negative (i.e., **w**$$_{p}\ge $$ **0**$$_{Jp\times 1})$$. Let z$$_{i,p}$$ denote the *i*th random variable in **z**$$_{p}$$ ($$i =$$ 1, 2, $$\cdots $$, $$J_{p})$$, which takes a value in $$z_{i,p}$$ $$\subset {\mathbb {R}}$$. Let w$$_{{i},{p}}$$ denote the *i*th element of **w**$$_{p}$$ ($$i =$$ 1, 2, $$\cdots $$, $$J_{p})$$.

### Proposition 1

*A* convex component has scores within the range of its indicators’ scores.

### Proof

Let m$_{\text {1}}\equiv$ *inf*{*inf* $z_{\text {1,}p}$, *inf* $z_{\text {2,}p}$, $\cdots$, *inf* $z_{Jp,p}$} and m$_{\text {2}}\equiv$ *sup*{*sup* $z_{\text {1,}p}$, *sup* $z_{\text {2,}p}$, $\cdots$, *sup* $z_{Jp,p}$}. Then, $\text {m}_{\text {1}} =$ ${\textrm{m}}_{1} \sum \limits _{i=1}^{J_{p} } {\textrm{w}_{i,p} } = \sum \limits _{i=1}^{J_{p} } {\textrm{m}_{1} {\textrm{w}}_{i,p} }$ $\le \upgamma _{{p}}= \sum \limits _{i=1}^{J_{p} } {\textrm{z}_{i,p} {\textrm{w}}_{i,p} } \le \sum \limits _{i=1}^{J_{p} } {{\textrm{m}}_{2} {\textrm{w}}_{i,p} } ={\textrm{m}}_{2} \sum \limits _{i=1}^{J_{p} } {{\textrm{w}}_{i,p} } =\text {m}_{\text {2}}$.

### Proposition 2

Each score of a convex component corresponds to a component score of an individual whose scores for indicators are all the same as the component score.

### Proof

Let g $\in {\mathcal {G}}_{p}$ denote a value of $\upgamma _{p}$, where ${\mathcal {G}}_{p} \subset {\mathbb {R}}$ is the set of all possible values $\upgamma _{{p}}$ can take in ${\mathbb {R}}$. If **z**$_{p} =$ [g, g, $\cdots$, g]$^\prime$ $=$ g**1**$_{Jp}$, then $\upgamma _{p}$ $=$** w**$_{p}\,^\prime$g**1**$_{Jp} =$ g.

### Proposition 3

The mean of a convex component is not fixed to zero but is determined by weights within the range of its indicators’ means.

### Proof

$$E(\upgamma _{p}) =$$ **w**$$_{p}\,^{\prime }E$$(**z**$$_{p}) =$$** w**$$_{p}\,^{\prime }{\varvec{\upmu }}_{p}$$. Thus, E($$\upgamma _{p})$$ varies depending on **w**$$_{p}$$ unless $${\varvec{\upmu }}_{p} =$$ **0**. Let $${\varvec{\upmu }}_{i,p}$$ denote the *i*th element of **u**$$_{p}$$. Let m$$_{\text {3}}\equiv $$ *inf*{$${{\upmu }}_{1,p}$$, $${{\upmu }}_{2,p}$$, $$\cdots $$, $${{\upmu }}_{Jp,p}$$} and m$$_{\text {4}}\equiv $$ *sup*{$${{\upmu }} _{1,p}$$, $${{\upmu }}_{2,p}$$, $$\cdots $$, $${{\upmu }}_{Jp,p}$$}. Then, m$$_{\text {3}} = {\textrm{m}}_{3} \sum \limits _{i=1}^{J_{p} } {{\textrm{w}}_{i,p} } =$$ $$\sum \limits _{i=1}^{J_{p} } {{\textrm{m}}_{3} {\textrm{w}}_{i,p} } \le E(\upgamma _{p}) ={{\textbf {w}}}_{p}\,^{\prime } {\varvec{\upmu }}_{p} = \sum \limits _{i=1}^{J_{p} } {{{\varvec{\upmu }} }_{i,p} {\textrm{w}}_{i,p} } \le \sum \limits _{i=1}^{J_{p} } {{\textrm{m}}_{4} {\textrm{w}}_{i,p} } ={\textrm{m}}_{4} \sum \limits _{i=1}^{J_{p} } {\text {w}_{i,p} } =$$ $$\text {m}_{\text {4}}$$.

### Proposition 4

The standard deviation of a convex component is not fixed to one but is determined by weights within the range from 0 to the maximum standard deviation of its indicators.

### Proof

var($$\upgamma _{p})^{\text {1/2}} =$$ (**w**$$_{p}\,^\prime $$var(**z**$$_{p})$$ **w**$$_{p})^{\text {1/2}}$$ $$=$$ (**w**$$_{p}\,^\prime $$ $$\varvec{\Sigma }_{p}$$ **w**$$_{p})^{\text {1/2}}$$, indicating that the standard deviation of $$\upgamma _{p}$$ depends on **w**$$_{p}$$. Let $$\sigma _{k,l,p}$$ denote the (*k*,*l*)th element of $$\varvec{\Sigma }_{p}$$. Let m$$_{\text {5}}\equiv $$ *sup*{$$\upsigma _{1{,}1p}$$, $$\upsigma _{2,2,p}$$, $$\cdots $$, $$\sigma _{Jp,Jp,p}$$}. Then, var($$\upgamma _{p})^{\text {1/2}} =$$ (**w**$$_{p}\,^{\prime }$$ $$\varvec{\Sigma }_{p}$$ **w**$$_{p})^{\text {1/2}}=$$ $$(\sum \limits _{k=1}^{J_{p} } {\sum \limits _{l=1}^{J_{p} } {{\textrm{w}}_{k,p} {\textrm{w}}_{l,p} \sigma _{k,l,p} } } )^{1/2}\le (\sum \limits _{k=1}^{J_{p} } {\sum \limits _{l=1}^{J_{p} } {{\textrm{w}}_{k,p} {\textrm{w}}_{l,p} } } {\textrm{m}}_{5} )^{1/2}=({\textrm{m}}_{5} \sum \limits _{k=1}^{J_{p} } {\sum \limits _{l=1}^{J_{p} } {{\textrm{w}}_{k,p} {\textrm{w}}_{l,p} } } )^{1/2} = ({\textrm{m}}_{5} \sum \limits _{k=1}^{J_{p} } {{\textrm{w}}_{k,p} (\sum \limits _{l=1}^{J_{p} } {{\textrm{w}}_{l,p} )} } )^{1/2}=({\textrm{m}}_{5} \sum \limits _{k=1}^{J_{p} } {{\textrm{w}}_{k,p} } )^{1/2}= {\textrm{m}}_{5}^{1/2}$$. Therefore, 0< *var*($$\upgamma _{p})^{\text {1/2}}\le $$ $$\textrm{m}_{5}^{1/2}$$.

### Proposition 5

Given a linearly independent set of indicators’ scores, a set of convex component scores has a unique set of weights that are nonnegative and summed up to one.

### Proof

Let **D**$$_{p}=$$ [**d**$$_{\cdot 1,{p}}$$,**d**$$_{\cdot 2,{p}}$$, $$\cdots $$,** d**$$_{\cdot {Jp},{p}}$$] denote a *N* by $$J_{p}$$ data matrix of **z**$$_{p}$$, where *N* is the total number of individuals and **d**$$_{\cdot {i},{p}}$$ is the score set of z$$_{i,p}$$ ($$i =$$ 1, 2, $$\cdots $$, $$J_{p})$$. Then, the score set of the *p*th convex component for *N* individuals, denoted by **g**$$_{\cdot {p}}$$, can be expressed as **g**$$_{\cdot {p}} =$$ **D**$$_{p}$$ **w**$$_{p}$$. Suppose that there exists a different set of weights, $${{\textbf {w}}}_{p+} =[w_{1,p+},w_{2,p+},\cdots , w_{Jp,p+}]^{\prime }$$, such that $${{\textbf {g}}}_{\cdot {p} }=$$ $${{\textbf {D}}}_{p}{{\textbf {w}}}_{p+}\,^\prime $$ and $${{\textbf {w}}}_{p+}\ne {{\textbf {w}}}_{p}$$. Then, $$0 = {{\textbf {g}}}_{\cdot p-} {{\textbf {g}}}_{{\cdot }{p}} = {{\textbf {D}}}_{p}{{\textbf {w}}}_{p}^{ }- {{\textbf {D}}}_{p}{{\textbf {w}}}_{p+} = {{\textbf {D}}}_{p}({{\textbf {w}}}_{p}^{ }- {{\textbf {w}}}_{p+}) = {{\textbf {d}}}_{\cdot 1,{p}}(w_{1,p}-{\textrm{w}}_{1,p+})+ {{\textbf {d}}}_{\cdot 2,{p}}({\textrm{w}}_{2,p}^{ }- {\textrm{w}}{_{2,p+}}) +\cdots + {{\textbf {d}}}_{\cdot {Jp},{p}}({\textrm{w}}_{Jp,p}- {\textrm{w}}{_{Jp,p+}})$$. By the assumption that {**d**$$_{\cdot 1,{p}}$$, **d**$$_{\cdot 2,{p}}$$, $$\cdots $$ ,  **d**$$_{\cdot {Jp},{p}}$$} is linearly independent, w$$_{1,p}=$$ w$$_{1,p+}$$, w$$_2{,p}^{ }=$$ w$$_{2,p+}$$, $$\cdots $$, w$$_{Jp,p} =$$ w$$_{Jp,p+}$$, which contradicts the assumption. By the definition of a convex component, **w**$$_{p}\,^\prime $$ **1**$$_{Jp} =$$ 1 and **w**$$_{p} \ge $$ **0**$$_{Jp\times 1}$$.

### Proposition 6

The path coefficient of a convex component on an outcome variable indicates the expected amount of change in the outcome variable for a unit change in each indicator of the convex component while holding other variables fixed.

### Proof

Let $$\upgamma _{q}$$ $$=$$b$$_{0,q} +$$ b$$_{p,q}\upgamma _{p} +$$ $${\varvec{\upalpha }}^\prime $$ **x**+ $$\upzeta _{q}$$ denote a structural model equation of the outcome variable $$\upgamma _{q}$$ on $$\upgamma _{p}$$ and a vector of covariates **x** for $$\upgamma _{q}$$, where b$$_{0,q}$$ is an intercept for $$\upgamma _{q}$$, b$$_{p,q}$$ is the path coefficient from $$\upgamma _{p}$$ to $$\upgamma _{q,}$$ $${\varvec{\upalpha }}$$ is a vector of path coefficients of **x**, and $$\upzeta _{q}$$ is an error term for $$\upgamma _{q}$$. As this model equation can be re-expressed as $$\upgamma _{q}$$ $$=$$ b$$_{0,q} +$$ b$$_{p,q}$$** w**$$_{p}\,^\prime $$ **z**$$_{p} +$$ $${\varvec{\upalpha }}\,^\prime $$ **x**+$$\upzeta _{q}$$, an expected change of $$\upgamma _{q}$$ for a one-unit change in every element of **z**$$_{p}$$ with the values of **x** fixed can be expressed as *E*((b$$_{0,q}+$$ b$$_{p,q}$$ **w**$$_{p}\,^\prime $$(**z**$$_{p} + $$ **1**$$_{p})\, + $$ $${\varvec{\upalpha }}\,^\prime $$ **x** + $$\upzeta _{q})$$− (b$$_{0,q}+$$ b$$_{p,q}$$ **w**$$_{p}\,^\prime $$ **z**$$_{p} +$$ $${\varvec{\upalpha }}\,^\prime $$**x **+ $$\upzeta _{q}))$$, which is equivalent to *E*(b$$_{p,q}$$ **w**$$_{p}\,^\prime $$(**z**$$_{p} + $$ **1**$$_{p})\, -$$ $$b_{p,q}$$ **w**$$_{p}\,^\prime $$ **z**$$_{p})$$ $$= E$$(b$$_{p,q}$$(**w**$$_{p}\,^\prime $$ **1**$$_{p}))$$ $$= E$$(b$$_{p,q}) =$$ b$$_{p,q}$$.

## Appendix 3. A Proof that the Optimization Function of Convex GSCA is Partially Scale-Invariant

Suppose that for each block of indicators that are on the same scale, the measurement scales are linearly transformed arbitrarily. Let ${{{\textbf {z}}}}_{new}$ $=$ ${\varvec{\Omega }} _{\text {z}}(\textbf{z}\, +\,\varvec{\lambda }\mathbf {)}$ denote a vector of rescaled indicators, where ${\varvec{\lambda }}$ is a *J* by 1 constant vector for relocation and ${\varvec{\Omega }}_{\text {z}}$ is a diagonal matrix for scalar multiplication for each indicator. This linear transformation of the measurement scales of indicators does not change the minimum value of the objective function (13) and the corresponding weight values, which can be proven as follows. Let ${\varvec{\upgamma }}_{new} = {{\textbf {W}}}^\prime {{{\textbf {z}}}}_{new}$ denote a vector of components defined with rescaled indicators. Let **e**$_{new}$ denote a vector of prediction errors for [${{{\textbf {z}}}}_{new}$; ${\varvec{\upgamma }}_{new}$]. Let **A**$_{new}$ denote a matrix of unstandardized loading and path coefficients for ${{{\textbf {z}}}}_{new}$. Let **a**$_{0,new}$ denote a vector of unstandardized intercepts for ${{{\textbf {z}}}}_{new}$. Let **O**$_{new}$ denote a diagonal matrix of penalty parameters for prediction errors given ${{{\textbf {z}}}}_{new}$. Let $\upomega _{p}$ is the scalar multiplier that is applied the *p*th block of indicators. Let ${\varvec{\Omega }} _{{\upgamma }} \equiv$ *blkdiag*($\upomega _{\text {1}}$, $\upomega _{\text {2}}$, $\cdots$, $\upomega _{P})$ and ${\varvec{\Omega }} \equiv$ *blkdiag*(${\varvec{\Omega }}_{\text {z}}$, ${\varvec{\Omega }}_{{\upgamma }})$.

When ${{\textbf {A}}}_{new} ={\varvec{\Omega }}_{{\upgamma }}^{{{-1}}}$ **A**${\varvec{\Omega }}$, **a**$_{0,new}$ $=$ ($\varvec{\lambda }^{\prime }$(**V** – **WA**)$\, +\,$ **a**$_{\text {0}}) {\varvec{\Omega }}$, and ${{{\textbf {O}}}}_{new}{\equiv }$ ${\varvec{\Omega }}^{{-1}}{{{\textbf {O}}}}$, the objective function (13) can be re-written as

A.3 $$\begin{aligned} &  f_{cvx}{(\textbf{W},\textbf{A},}{{{\textbf {a}}}}_{\text {0}})\nonumber \\ &  \quad =tr{(\textbf{O}}E{(\textbf{ee}')\textbf{O})}\nonumber \\ &  \quad =E(SS{((\textbf{z}'\textbf{V}-(\textbf{z}'\textbf{WA}}+{{{\textbf {a}}}}_{{0}}{'))\textbf{O})}\nonumber \\ &  \quad =E(SS{((\textbf{z}'(\textbf{V}-\textbf{WA})}- {{{\textbf {a}}}}_{{0}}{')\textbf{O})}\nonumber \\ &  \quad =E(SS{((\textbf{z}'+\varvec{\lambda }^{\prime }-\varvec{\lambda }'}\mathbf {)}{\varvec{\Omega }}_{\text {z}}{\varvec{\Omega }}_{\text {z}}^{{-1}}{(\textbf{V}-\textbf{WA})}-{{{\textbf {a}}}}_{{0}}{') \varvec{\Omega }}{\varvec{\Omega }}^{{-1}}{{{\textbf {O}}})}\nonumber \\ &  \quad =E(SS{((\textbf{z}+\varvec{\lambda }}\mathbf {)}'{\varvec{\Omega }}_{\text {z}}({\varvec{\Omega }}_{\text {z}}^{{-1}}{{{\textbf {V}}}-}{\varvec{\Omega }}_{\text {z}}^{{-1}}{{\textbf {WA}})\varvec{\Omega }-(\varvec{\lambda }'(\textbf{V}-\textbf{WA})} + {{{\textbf {a}}}}_{{0}}{') \varvec{\Omega })}{\varvec{\Omega }}^{{-1}}{{{\textbf {O}}})}\nonumber \\ &  \quad =E(SS({{{\textbf {z}}}}_{new}'([{\varvec{\Omega }}_{\text {z}}^{{-1}} ,{\varvec{\Omega }}_{\text {z}}^{{-1}}{{{\textbf {W}}}]\varvec{\Omega }}-{\varvec{\Omega }}_{\text {z}}^{{-1}}{{\textbf {WA}} \varvec{\Omega })}-{{{\textbf {a}}}}_{\text {0,}new}{'))}{{{\textbf {O}}}}_{new})\nonumber \\ &  \quad =E(SS({{{\textbf {z}}}}_{new}\,^{\prime }([{{{\textbf {I}}}}_{J},{{{\textbf {W}}}]}{\varvec{\Omega }}^{{-1}}{\varvec{\Omega }-\textbf{W}}{\varvec{\Omega }}_{{\upgamma }}^{{-1}}{{{\textbf {A}}}{\varvec{\Omega }} )}-{{{\textbf {a}}}}_{{0,}new}{'))}{{{\textbf {O}}}}_{new})\nonumber \\ &  \quad =E(SS({{{\textbf {z}}}}_{new}{'(\textbf{V}-\textbf{W}}{{{\textbf {A}}}}_{new})-{{{\textbf {a}}}}_{{0,}new}{'))}{{{\textbf {O}}}}_{new})\nonumber \\ &  \quad =E(SS({{{\textbf {z}}}}_{new}{'\textbf{V}-(}{{{\textbf {z}}}}_{new}{'\textbf{W}}{{{\textbf {A}}}}_{new}+{{{\textbf {a}}}}_{{0,}new}{'))}{{{\textbf {O}}}}_{new})\nonumber \\ &  \quad =tr({{{\textbf {O}}}}_{new}E({{{\textbf {e}}}}_{new}{{{\textbf {e}}}}_{new}{')}{{{\textbf {O}}}}_{new}) \nonumber \\ &  \quad =f_{cvx}{(\textbf{W},}{{{\textbf {A}}}}_{new},{{{\textbf {a}}}}_{{0,}new}{).} \end{aligned}$$

The seventh equality in (A.3) holds because ${\varvec{\Omega }}_{\text {z}}^{{-1}}{{{\textbf {W}}}}$ $=$ ${{{\textbf {W}}}}{\varvec{\Omega }}_{{\upgamma }}^{{-1}}$.

## Appendix 4. A Description of GSCA $_{{cvx}}$’s ALS Algorithm

The objective function (13) can be re-written as

A.4 $$\begin{aligned} &  f_{cvx}{(\textbf{W,A}},{{{\textbf {a}}}}_{{0}})\nonumber \\ &  \quad =E(SS{((\textbf{z}'(\textbf{V}-\textbf{WA})}-{{{\textbf {a}}}}_{{0}}{')\textbf{O}))}\nonumber \\ &  \quad =E{(SS((\textbf{z}-\varvec{\upmu })'\textbf{LO}-(}{{{\textbf {a}}}}_{{0}}^{\prime }-{\varvec{\upmu }'\textbf{L})\textbf{O}))}\nonumber \\ &  \quad =E(tr{(((\textbf{z}-\varvec{\upmu })'\textbf{LO}-(}{{{\textbf {a}}}}_{{0}}{'-\varvec{\upmu }'\textbf{L})\textbf{O})'((\textbf{z}-\varvec{\upmu })'\textbf{LO}-(}{{{\textbf {a}}}}_{{0}}^\prime -\varvec{\upmu }^\prime \textbf{L})\textbf{O})))\nonumber \\ &  \quad =tr{(\textbf{OL}'\varvec{\Sigma \textrm{LO}})-2}tr{(\textbf{O}(}{{{\textbf {a}}}}_{{0}}^{\prime }-{\varvec{\upmu }'\textbf{L})'}E{(\textbf{z}-\varvec{\upmu })'\textbf{LO})\,+\,}SS{((}{{{\textbf {a}}}}_{{0}}^{\prime }-{\varvec{\upmu }'\textbf{L})\textbf{O}))}\nonumber \\ &  \quad =tr{(\textbf{OL}'\varvec{\Sigma \mathrm{\textrm{LO}}})\, +\, }SS{((}{{{\textbf {a}}}}_{{0}}^{\prime }-{\varvec{\upmu }'\textbf{L})\textbf{O})),} \end{aligned}$$

where **L** $$\equiv $$ **V** – **WA**. Let **S** denote the positive definite sample covariance matrix of indicators and $${\widehat{{\textbf {O}}}}$$ denote the sample analogy of **O**. As $${\varvec{\Sigma }}$$, $${\varvec{\upmu }} $$, and **O** are typically not available, GSCA$$_{cvx} $$ replaces $$\varvec{\Sigma }$$, $${\varvec{\upmu }} $$, and **O** in (A.4) with **S**, $${\hat{{\varvec{\upmu }}}}$$, and $${\widehat{{\textbf {O}}}}$$, respectively, as follows,

A.5 $$\begin{aligned} &  f_{cvx}^{*}{(\textbf{W,A,}}\, {{{\textbf {a}}}}_{{0}})\nonumber \\ &  \quad =tr({\widehat{{\textbf {O}}}}{{{\textbf {L}}}'\textbf{SL}}{\widehat{{\textbf {O}}}})+SS(({{{\textbf {a}}}}_{{0}}{'}-{\hat{{\varvec{\upmu }}}}'\textbf{L}){\widehat{{\textbf {O}}}}), \end{aligned}$$

and applies the ALS algorithm to find the minimum point of (A.5) with respect to **W**, **A**, and **a**$_{{{{\textbf {0}}}}}$ subject to ${{{\textbf {w}}}}_{p}\,^{\prime }$ **S**$_{p}{{{\textbf {w}}}}_{p}$ $=$ 1 or **1**$_{Jp}\,^{\prime }{{{\textbf {w}}}}_{p} =$ 1 ($p =$ 1, 2, $\cdots$, *P*), where ${{{\textbf {S}}}}_{p}$ is an $J_{p}$ by $J_{p}$ sample covariance matrix of **z**$_{p}$.

The proposed ALS algorithm begins by assigning random initial values to **A** and repeats two steps until convergence. In the first step, **W** and **a**$_{{0}}$ are updated with **A** fixed. By solving $\frac{1}{2}\frac{\partial f_{cvx}^{*} }{\partial {{\textbf {a}}}_{0} }=0$, the least square estimates of **a**$_{{0}}$ given **W** and **A** can be obtained as

A.6 $$\begin{aligned} {\hat{{\textbf {a}}}}_{0}\, {=}\, \hat{{\varvec{\upmu }}}{(\textbf{V}-\textbf{WA})} \end{aligned}$$

This implies that the least squares estimate of **a**$_{{0}}$ can be expressed as a function of **W** and **A**. In other words, if we can find the least square estimate of **W** given **A** under the constraint (A.6), we can obtain ${{\hat{{\textbf {a}}}}}_{0}$ as well by (A.6). Inserting (A.6) into (A.5) makes (A.5) be simplified as

A.7 $$\begin{aligned} &  f_{cvx}^{*}{(\textbf{W,A})}\nonumber \\ &  \quad =tr({\widehat{{\textbf {O}}}}{(\textbf{V}-\textbf{WA})'\textbf{S}(\textbf{V}-\textbf{WA})}{\widehat{{\textbf {O}}}})\nonumber \\ &  \quad =N^{{-1}}SS({{{\textbf {D}}}}_{ct}{(\textbf{V}-\textbf{WA})}{\widehat{{\textbf {O}}}}), \end{aligned}$$

where ${{{\textbf {D}}}}_{ct} \equiv$ **D** – ${{{\textbf {1}}}}_{N}{\hat{{\varvec{\upmu }}}}'$. Let ${\widehat{{\textbf {O}}}}_{\text {Y}}$ denote a *T* by $T_{\text {Y}}$ matrix consisting of all nonzero columns of ${\widehat{{\textbf {O}}}}$, where $T \equiv P + J$ and $T_{\text {Y}}$ is the number of dependent variables in the model. Let **I**$_{{0}}$ $\equiv$ [**I**$_{J}$, **0**$_{{J}\times {P}}$] and ${{{\textbf {A}}}}_{\text {I}} \equiv$ **A** – ${[}{{{\textbf {0}}}}_{P{\times }J},{{{\textbf {I}}}}_{P}{]}$. Let **W**$_{{-}p}$ denote a *J* by (*P* – 1) matrix formed by the columns of **W** except for its *p*th column. Let ${{{\textbf {A}}}}_{\text {I,--}p}$ denote a (*P* – 1) by *T* matrix formed by the rows of **A**$_{\text {I}}$ except for its *p*th row and ${{{\textbf {a}}}}_{p}$ denote a row vector whose entries are the non-zero elements of the *p*th row of ${{{\textbf {A}}}}_{\text {I,--}p}$ corresponding to **w**$_{p}$. Let *vec*() denote an operator that returns a column vector obtained by stacking the columns of input matrixvertically. Given **A**, (A.7) can be re-expressed as

A.8 $$\begin{aligned} &  f_{cvx}^{*}({{{\textbf {w}}}}_{p}{;\textbf{A}},{{{\textbf {W}}}}_{{-}p})\nonumber \\ &  \quad =N^{{-1}}SS({{{\textbf {D}}}}_{ct}{(\textbf{V}-\textbf{WA})}{\widehat{{\textbf {O}}}}_{\text {Y}})\nonumber \\ &  \quad =N^{{-1}}SS({{{\textbf {D}}}}_{ct}(\left[ {{{\textbf {I}}}}_{J},{{{\textbf {0}}}}_{J{\times }P} \right] {+[}{{{\textbf {0}}}}_{J{\times }J},{{{\textbf {W}}}]-\textbf{WA})}{\widehat{{\textbf {O}}}}_{\text {Y}})\nonumber \\ &  \quad =N^{{-1}}SS({{{\textbf {D}}}}_{ct}{{(\textbf{I}}}_{{0}}- \textbf{W}{{{\textbf {A}}}}_{\text {I}}){\widehat{{\textbf {O}}}}_{\text {Y}})\nonumber \\ &  \quad =N^{{-1}}SS({{{\textbf {D}}}}_{ct}{{(\textbf{I}}}_{{0}}-\textbf{W}_{{-}p}{{{\textbf {A}}}}_{\text {I},-p}\, {-}\, {{{\textbf {w}}}}_{p}{{{\textbf {a}}}}_{p}){\widehat{{\textbf {O}}}}_{\text {Y}})\nonumber \\ &  \quad =N^{{-1}}SS(vec({{{\textbf {D}}}}_{ct}{{(\textbf{I}}}_{{0}}-{{{\textbf {W}}}}_{{-}p}{{{\textbf {A}}}}_{\text {I,}-p}){\widehat{{\textbf {O}}}}_{\text {Y}}{)-((}{{{\textbf {a}}}}_{p}{\widehat{{\textbf {O}}}}_{\text {Y}}{)'\, \otimes }\,{{{\textbf {D}}}}_{ct}){{{\textbf {w}}}}_{p}))\nonumber \\ &  \quad =N^{{-1}}SS({\varvec{\uppsi }}_{{1}}-{\varvec{\Xi }}_{{1}}{{{\textbf {w}}}}_{p})), \end{aligned}$$

where ${\varvec{\uppsi }}_{{1}}$ $\equiv$ *vec*(${{{\textbf {D}}}}_{ct}$(**I**$_{{0}}$ –** W**$_{-p}$ **A**$_{\textrm{I},-{p}}){\widehat{{\textbf {O}}}}_{\text {Y}})$ and ${\varvec{\Xi }}_{\text {1}}\equiv$ $({{{\textbf {a}}}}_{p}{\widehat{{\textbf {O}}}}_{\text {Y}})'\otimes {{{\textbf {D}}}}_{ct}$.

If $\upgamma _{p}$ is standardized component, the unstandardized least square estimate of **w**$_{p}$ is obtained by

A.9 $$\begin{aligned} {{\widehat{{\textbf {w}}}}_{p{*}}{=(}{\varvec{\Xi }}_{{1}}'{\varvec{\Xi }}_{{1}})}^{{-1}}{\varvec{\Xi }}_{{1}}{\varvec{\uppsi }}_{{1}}. \end{aligned}$$

Then, the standardized least square estimate of **w**$_{p}$ is obtained by ${\hat{{\textbf {w}}}}_{p} =$ (${\hat{{\textbf {w}}}}_{p{*}}\,^{\prime }{{{\textbf {S}}}}_{p}{\hat{{\textbf {w}}}}_{p{*}})^{{1/2}}$ such that ${\hat{{\textbf {w}}}}_{p}$ can satisfy ${\hat{{\textbf {w}}}}_{p}\,^{\prime }{{{\textbf {S}}}}_{p}{\hat{{\textbf {w}}}}_{p} =$ 1. If every element of ${\hat{{\textbf {w}}}}_{p}$ is forced to be positive, finding ${\hat{{\textbf {w}}}}_{p}$ that minimizes (A.8) becomes a well-known *nonnegative least squares problem* (NNLS; Lawson & Hanson, 1974, Chapter 23), which should be solved numerically. For instance, the function *lsqnonneg* in MATLAB or the *nnls* package in R can be utilized under this condition.

If $\upgamma _{p}$ is a convex component, the ALS algorithm finds the solution for (A.8) subject to **1**$_{Jp}\,^{\prime }$ **w**$_{p} =$ 1. This minimization is a linearly constrained least squares problem (Boyd & Vandenberghe, 2018, Chapter 16). As the product of the ranks of two matrices equals to the rank of the Kronecker product of the two matrices and ${{{\textbf {a}}}}_{p}{\hat{{\textbf {O}}}}_{\text {Y}}$ has one row, $({{{\textbf {a}}}}_{p}{\hat{{\textbf {O}}}}_{\text {Y}})'\otimes {{{\textbf {D}}}}_{ct}$ has linearly independent columns, thereby having the columns of $$\left[ {\begin{array}{ll}\varvec{\Xi }_1& \\ {{\textbf {1}}}_{Jp}'& \\ \end{array} } \right] $$ are also linearly independent. Thus, there exists ${\varvec{\updelta }}$ satisfying

A.10 $$\begin{aligned} \left[ {\begin{array}{ll}\varvec{\Xi }_1 ^{\prime }\varvec{\Xi }_1& {{\textbf {1}}}_{Jp}\\ {{\textbf {1}}}_{Jp} ^\prime & 0\\ \end{array} } \right] \left[ {\begin{array}{ll}{{\textbf {w}}}_p& \\ \varvec{\updelta }& \\ \end{array} } \right] =\left[ {\begin{array}{ll}\varvec{\Xi }_1 & ^{\prime }\varvec{\Psi }_1\\ 1& \\ \end{array} } \right] , \end{aligned}$$

where $$\left[ {\begin{array}{ll}\varvec{\Xi }_1 ^{\prime }\varvec{\Xi }_1& {{\textbf {1}}}_{Jp}\\ {{\textbf {1}}}_{Jp} ^\prime & 0\\ \end{array} } \right] $$ is invertible. Let $${\varvec{\Xi }}_{{2}} \equiv \left[ {\begin{array}{ll}\varvec{\Xi }_1 ^{\prime }\varvec{\Xi }_1& {{\textbf {1}}}_{Jp}\\ {{\textbf {1}}}_{Jp} ^\prime & 0\\ \end{array} } \right] $$ and $${\varvec{\uppsi }}_{{2}}$$$$\equiv \left[ {\begin{array}{ll}\varvec{\Xi }_1& ^{\prime }\varvec{\Psi }_1\\ 1& \\ \end{array} } \right] $$. Then, $${\hat{{\textbf {w}}}}_{p}$$ can be obtained by the first $$J_{p}$$ entries of $${\varvec{\Xi }}_{{2}}^{-1}{\varvec{\uppsi }}_{{2}}$$, from which $${\hat{{\textbf {W}}}}$$ is updated. If **w**$$_{p}$$ is forced to be positive, minimizing (A.8) becomes a quadratic programming problem with a linear constraint and an inequality constraint (e.g., Floudas & Visweswaran, 1995; Frank & Wolfe, 1956). This problem does not have closed-form solution. Instead, it can be solved numerically via interior point methods (e.g., Altman & Gondzio, 1999; Vanderbei & Carpenter, 1993). For instance, the function *lsqlin* in MATLAB or the *quadprog* package in R can be utilized to minimize (A.8) numerically. This process repeats for every **w**$$_{p}$$ ($$p =$$ 1, 2, $$\cdots $$, *P*). Then $${\hat{{\textbf {a}}}}_{0}$$ is updated by (A.6).

In the second step, **A** and **a**$_{{{{\textbf {0}}}}}$ are updated with **W** fixed. Given **W**, (A.8) can be re-expressed as

A.11 $$\begin{aligned} &  f_{cvx}^{*}{(\textbf{A};\textbf{W})}\nonumber \\ &  \quad =N^{{-1}}SS(vec({{{\textbf {D}}}}_{ct}{{{\textbf {V}}}}{\hat{{\textbf {O}}}}_{\text {Y}})-({\hat{{\textbf {O}}}}_{\text {Y}}{'\otimes (}{{{\textbf {D}}}}_{ct}{{{\textbf {W}}}))}vec{(\textbf{A}))}\nonumber \\ &  \quad =N^{{-1}}SS(vec({{{\textbf {D}}}}_{ct}{{{\textbf {V}}}}{\hat{{\textbf {O}}}}_{\text {Y}}{)} - {\varvec{\Xi }}_{{3}}{\varvec{\uprho }),} \end{aligned}$$

where ${\varvec{\Xi }}_{{3}}$ is the matrix formed by the columns of$({\hat{{\textbf {O}}}}_{\text {Y}}{'\otimes (}{{{\textbf {D}}}}_{ct}{{{\textbf {W}}}))}$ corresponding to the nonzero elements in *vec*(**A**), and ${\varvec{\uprho }}$ is the column vector of the nonzero elements of *vec*(**A**). Then, the value of ${\varvec{\uprho }}$ that minimizes (A.11) is obtained by

A.12 $$\begin{aligned} {\varvec{\uprho }}\quad = ({\varvec{\Xi }}_{{3}}'{\varvec{\Xi }}_{{3}})^{{-1}}{\varvec{\Xi }}_{{3}}'vec({{{\textbf {D}}}}_{ct}{{{\textbf {V}}}} {\hat{{\textbf {O}}}}_{\text {Y}}) \end{aligned}$$

from which the non-zero elements of ${\hat{{\textbf {A}}}}$ are updated. Then ${\hat{{\textbf {a}}}}_{0}$ is updated by (A.6).

## Appendix 5. A Procedure for Deriving the Population Covariance Matrix of Indicators from the Prescribed Parameter Values of the GSCA Model with Convex Components

The proposed procedure imitates the one suggested by Cho and Choi’s (2020) one, while simply replacing standardized components and correlation matrix of indicators with convex components and covariance matrix of indicators, respectively. Let **c**$$_{p}$$ is a $$J_{p} $$ by 1 vector of loadings for **z**$$_{p}$$ Let $$\varvec{\upxi }_{p}$$ is a $$J_{p} $$ by 1 vector of error terms for **z**$$_{p}$$. Let $${\varvec{\Phi }}_{std} $$ denote a *P* by *P* correlation matrix of components. Let $${\varvec{\Delta }} $$ denote a *P* by *P* diagonal matrix whose *p*th entry is the standard deviation of $$\upgamma _{p}$$, denoted by $$\upphi _{p}$$. Let $${\varvec{\Theta }} =$$ *blkdiag*($${\varvec{\Theta }}_{{1}}$$, $${\varvec{\Theta }} _{{2}}$$, $$\cdots $$, $${\varvec{\Theta }} _{P})$$ denote a *J* by *J* covariance matrix of errors in the measurement model, where $${\varvec{\Theta }}_{p}$$ is a $$J_{p}$$ by $$J_{p}$$ covariance matrix of errors for the *p*th block of indicators. Let $${\varvec{\uptau }} =$$ [$$\uptau _{{1}}$$, $$\uptau _{{2}}$$, $$\cdots $$, $$\uptau _{P}\,^\prime $$] denote a *P* by 1 vector of component means.

Given the prescribed values of $${\varvec{\Sigma }}_{p}$$, $${\varvec{\upmu }} _{p}$$, and $${\varvec{\Phi }}_{std}(p =$$ 1, 2, $$\cdots $$, *P*), **w**$$_{p}$$ is obtained by **w**$$_{p} =$$ ($${\varvec{\Sigma }} _{p}^{{-1/2}}$$ **u**$$_{1p})$$ / (**1**$$_{p}\,^{\prime }$$ $${\varvec{\Sigma }} _{p}^{{-1/2}}$$ **u**$$_{1p})$$, where **u**$$_{1p}$$ is the eigenvector corresponding to the largest eigenvalue of $${\varvec{\Sigma }}_{p}$$, indicating that **w**$$_{p}$$ maximizes the sum of explained variances of **z**$$_{p}$$ given $${\varvec{\Sigma }}_{p}$$ subject to **1**$$_{p}\,^{\prime }$$ **w**$$_{p} =$$ 1. Then, $$\uptau _{p}$$ and $$\upphi _{p}$$ are calculated as $$\uptau _{p} =$$ **w**$$_{p}\,^{\prime }$$ $${\varvec{\upmu }} _{p}$$ and $$\upphi _{p} =$$ **w**$$_{p}\,^{\prime }$$ $${\varvec{\Sigma }} _{p}$$ **w**$$_{p}$$, respectively, based on which **c**$$_{p}$$ are obtained by **c**$$_{p} = \upphi _{p}^{{-2}} {\varvec{\Sigma }}_{p}$$ **w**$$_{p}$$, implying that **c**$$_{p}$$ is a vector of least-square loading values of **z**$$_{p}$$ on $$\upgamma _{p}$$. In turn, $${\varvec{\Theta }}_{p}$$ is obtained by $${\varvec{\Theta }}_{p}=$$ (**I**$$_{Jp}$$ – **c**$$_{p}$$ **w**$$_{p}\,^{\prime }$$) $${\varvec{\Sigma }} _{p}$$(**I**$$_{Jp}$$ – **w**$$_{p}$$ **c**$$_{p}\,^{\prime }$$). Then, all the block-diagonal elements of **W**, **C**, and $${\varvec{\Theta }} $$ can be filled in with **w**$$_{p}$$, **c**$$_{p}$$, and $${\varvec{\Theta }}_{p}(p =$$ 1, 2, $$\cdots $$, *P*), respectively. Also, $${\varvec{\uptau }} $$ is computed by $${\varvec{\uptau }} =$$ **W**$$^{\prime }$$ $${\varvec{\upmu }} $$ and then, $${{\textbf {c}}}_0$$ is calculated as $${{\textbf {c}}}_0$$ $$=$$ $${\varvec{\upmu }} $$ – **C**$$^{\prime }$$ $${\varvec{\uptau }}$$. Next, $${\varvec{\Phi }}$$ is derived by $${\varvec{\Phi }} =$$ $$\varvec{\Delta }{\varvec{\Phi }}_{std} \varvec{\Delta }$$. Finally, we obtain $${\varvec{\Sigma }}$$ by $${\varvec{\Sigma }}=\,\, $$**C**$$^{\prime }$$ $$\varvec{\Phi }$$ **C**  + $${\varvec{\Theta }} $$. A more detailed explanation on each step of this procedure can be found in Cho and Choi (2020).

## Footnotes

1 The MATLAB code is available at https://osf.io/y75kg/?view_only=0d02aea6aaaa4aa29d405172544aae7d.
