# Correction to: Generalized Structured Component Analysis Accommodating Convex Components: A Knowledge-Based Multivariate Method with Interpretable Composite Indexes

**DOI:** 10.1007/s11336-024-10001-w

**Published:** 2024-09-14

**Authors:** Gyeongcheol Cho, Heungsun Hwang

**Affiliations:** 1 Department of Psychology, The Ohio State University, 1827 Neil Avenue, Columbus, OH 43210 USA; 2 Department of Psychology, McGill University, Montreal, Canada

**Correction to: Psychometrika vol. 89, no. 1, 241–266**

10.1007/s11336-023-09944-3

In this article the below section numbers are incorrectly placed.

The section 1.3 should be replaced to section 2.

The section 2 should be replaced to section 3.

The sub section 2.1, 2.2 and 2.3 should be replaced to 3.1, 3.2 and 3.3.

The section 3 should be replaced to section 4.

The section 4 should be replaced to section 5.

The section 5 should be replaced to section 6.

In the introduction section in paragraph 7 the citation “sect. 1” to be replaced to “sect. 2”.

In the section 1.2 in paragraph 2 the citation “sect. 4” to be replaced to “sect. 5”.

In the section 1.2. in paragraph 2 the citation "sect. 1" to be replaced to "the introduction".

The original article has been corrected.

